# Flexible Metal–Organic Frameworks for Gas Handling Operations of CO_2_ and Its Isotopes: Mechanisms, Regulation Strategies and Potential Applications

**DOI:** 10.1007/s40820-026-02218-4

**Published:** 2026-05-18

**Authors:** Na Geng, Ningyu Liu, Sai Chu, Yongjian Huang, Lu Bai, Ming-Shui Yao, Yangyang Guo, Tingyu Zhu

**Affiliations:** 1 State Key Laboratory of Mesoscience and Process Engineering, Institute of Process Engineering, Chinese Academy of Sciences, Beijing, 100190 People’s Republic of China; 2 School of Chemical Engineering, University of Chinese Academy of Sciences, Beijing, 100049 People’s Republic of China; 3 HBIS Group Co., Ltd., Shijiazhuang, 050023 People’s Republic of China

**Keywords:** Carbon capture, Flexible metal–organic frameworks, Adsorption mechanisms, Flexibility regulate, Vacuum pressure swing adsorption

## Abstract

Global warming and the utilization of greenhouse gases have become a matter of worldwide concern. Porous adsorbents have emerged as core materials for effectively capturing CO_2_ and its isotopes. Flexible metal–organic frameworks (MOFs) stand out from traditional rigid adsorbents due to their unique structural flexibility and dynamic behavior. Owing to these characteristics, flexible MOFs have emerged as highly promising materials for CO_2_ adsorption and separation. Moreover, the high working capacity and excellent selectivity of flexible MOFs confer significant advantages for CO_2_ capture applications. They are expected to reduce energy consumption during adsorption–desorption cycles, positioning them as a promising new adsorbent. This review offers an overview of the dynamic behaviors of CO_2_ adsorption by flexible MOFs. Furthermore, we summarized the cutting-edge achievements in adjusting gating pressure, adsorption hysteresis loops, and CO_2_ affinity through ligand engineering, regulating the metal node, and functionalizing the pore environment. The challenges encountered with the material during actual carbon capture were discussed. Finally, an in-depth prospect is provided to promote the application of these materials in low-carbon energy and the high-value utilization of CO_2_ and its isotopes.

## Highlights

- An overview of CO_2_ adsorption mechanisms in flexible metal-organic frameworks (MOFs) is presented from dynamic structure transformation and host-guest interactions.
- Strategies of flexible regulation in the metal node, ligand, and pore functionalization are summarized.
- The key challenges of vacuum pressure swing adsorption matching, industrial applications, and future opportunities of flexible MOFs are proposed.

## Graphical abstract

**Figure:**
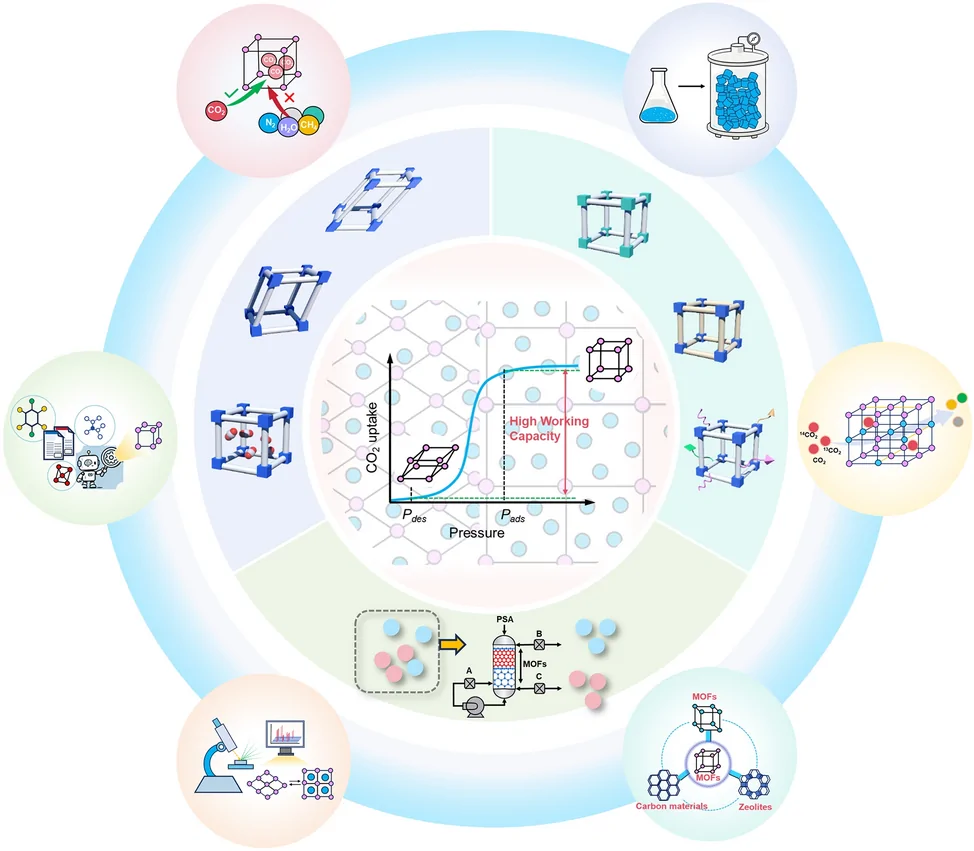

## Introduction

Carbon dioxide (CO_2_) is not only one of the primary greenhouse gases (GHG) in the atmosphere contributing to global warming, but also the basic material for high-value products, such as alcohols, polymers, isotopic urea, etc. Over the past 60 years, there has been a marked increase in CO_2_ concentrations, rising from 310 ppm in 1960 to 419 ppm in 2023 [1, 2], mainly due to fossil fuel use and human activities [3, 4]. As a result, developing carbon capture technologies, particularly Carbon Capture, Utilization, and Storage (CCUS), is essential for reducing industrial CO_2_ emissions, with carbon capture focusing on gases from sources like flue gas [5–7].

In recent decades, CO_2_ capture technology has been continuously advanced, involving advanced separation strategies, specifically liquid-phase absorption, solid-phase adsorption, and membrane separation technologies [2, 8–10]. They can realize CO_2_ and/or isotope capture under different conditions [11, 12]. Among them, the solid-phase adsorption, especially vacuum pressure swing adsorption (VPSA) technology, is widely considered for large-scale handling operations due to its operational simplicity and low energy consumption [13, 14]. Nevertheless, the design and controlled preparation of advanced adsorbent materials remain significant challenges. Improving the working capacity of adsorbents is essential for advancing CO_2_ capture via VPSA. Compared with the amine solution absorption method, porous materials are attracting attention because of their inherent potential in reducing energy consumption. Activated carbon and zeolites exhibit certain cost advantages, but they still face limitations such as susceptibility to desorption, low CO_2_ adsorption enthalpy, or excessive hydrophilicity [15, 16]. In the 1990s, driven by the proof-of-concept that coordination networks can be porous [19], the field of coordination networks and porous materials entered an active period, during which a large number of new framework structures were developed. These materials were later defined as porous coordination polymers (PCPs) or metal–organic frameworks (MOFs) [17, 18]. The pore shape, pore size, and other adjustable surface properties of these MOFs can be well regulated. It is confirmed that these frameworks and pores can realize the adsorption of small gas molecules at room temperature [19, 20]. The rich characteristics of MOFs, including high specific surface area, adjustable pore size, and diversified functional modifications, make MOFs show more possibilities in carbon capture than carbon materials and zeolites [21–23].

Since 1998, MOFs have been divided into three categories. The first-generation materials collapsed upon removing the guest molecules [24]. The second-generation materials can maintain a stable, robust structure before and after adsorption. The third-generation material is flexible MOFs with a dynamic porous frame structure, also known as “soft porous crystal”. Flexible MOFs constitute a class of porous materials that uniquely integrate long-range crystalline order with dynamic structural responsiveness. These materials are characterized by their ability to exist in two or more distinct phases, exhibiting reversible transitions between these states while maintaining permanent porosity. The intraframework interactions influence dynamicity, especially in compounds featuring integrated self-assembly of coordination nets. Owing to their strong interactions with the MOF skeleton, guest molecules accommodated in the void spaces during the synthesis process often play a key role in triggering framework flexibility. Likewise, the unique structural feature of flexible MOFs is the reversible transformation or dynamic change of structure when stimulated by external factors (e.g., pressure, temperature, light, electricity or magnetism, solvent, guest, etc.), which can also be classified into global and local flexibility [25–27]. Global flexibility is often accompanied by the expansion or contraction of ordered structures and subnetwork displacement, involving the cooperative motion of the entire crystal structure. Local flexibility typically involves restricted intramolecular motion or conformational adjustments of some units within a framework, including linker rotation and side chain movement. There are various terms to describe flexible MOFs, such as “flexible”, “soft”, “dynamic”, “stimuli-responsive”, and so on. Crucially, the flexibility of this porous framework enables a structural transition that is synergistically coupled with electron transfer and spin transitions.

Generally, in gas adsorption and separation, rigid adsorbents rely on diffusion effect or size matching to improve the recognition ability by enhancing the affinity for the target gas. Nonetheless, their ability to selectively recognize molecules in more complex gas mixtures is still limited. Unlike most rigid MOFs with fixed pore structures that rely on size-exclusion mechanisms, flexible MOFs dynamically modulate their pore environments through structural adaptability, enabling more precise molecular recognition. This active molecular recognition achieves superior selectivity in applications, such as CO_2_/N_2_ separation, isotope separation, C_2_H_2_/CO_2_ separation, and isomer separation [28]. The CO_2_ adsorption isotherm of traditional rigid porous adsorbents usually shows a type I isotherm [29, 30]. However, for many flexible MOFs, due to the phase transition, the adsorption of CO_2_ or other gases often shows “S-shape” or “step-type” isotherms [25, 31, 32]. This is due to the “breathing” or “gate opening” effect. This adsorption–desorption isotherm was reclassified as type F-I to F-V [33]. Generally, when the concentration or adsorption pressure of the gas molecules is low, the adsorption amount approaches zero. Upon reaching the critical pressure, the interaction energy between the guest and framework becomes sufficient to overcome the activation energy barrier, causing a cooperative transformation in the framework structure. Consequently, the adsorption amount increases abruptly. This phenomenon originates from the reversible switching between the “closed-pore phase” (cp) and the “open-pore/large-pore phase” (op/lp) within the framework’s pore structure. Between these two extreme states, there also exist metastable intermediate phases or narrow pore phases (np) in varying quantities [34]. It is worth noting that, during desorption, it must overcome an energy barrier from the open phase to the closed phase [35]. This causes gas uptake during desorption to exceed that during adsorption at the same pressure, producing a hysteresis loop in the adsorption–desorption isotherms. The multistable property enables flexible MOFs to selectively adsorb and separate gases by adaptively adjusting their structure in response to different environments and gas molecules [36, 37].

**Fig. 1:**
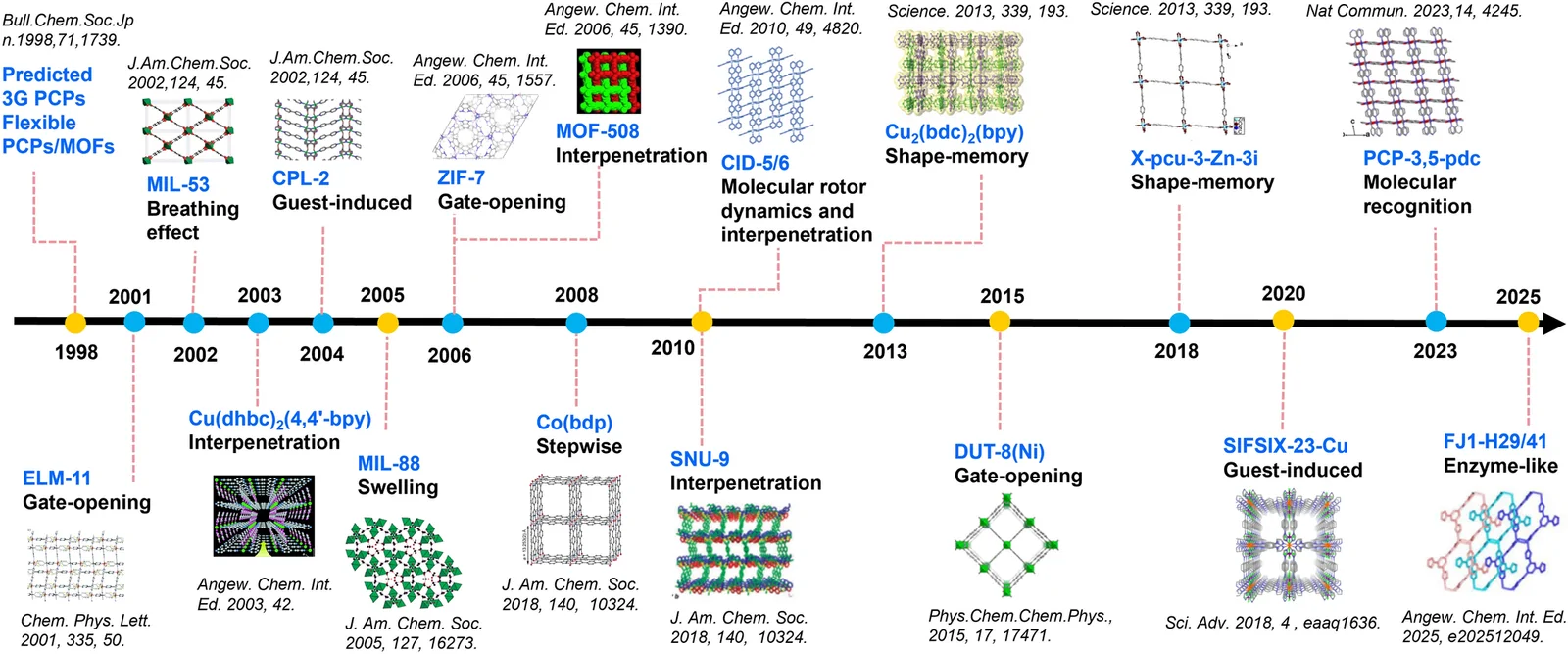
Timeline of some typical flexible MOFs for CO_2_ adsorption

For CO_2_ adsorption or separation processes, adsorbents with great selectivity and high working capacity are key to achieving efficient adsorption and separation. Flexible MOFs (Fig. 1), with their unique structural dynamic response, provide an ideal solution for meeting these two requirements simultaneously [38]. Firstly, the porous framework of flexible MOFs can provide adequate adsorption sites through structural expansion, thereby achieving a high CO_2_ working capacity. Alternatively, its core advantage lies in a selective “gating” mechanism—targeting mixed systems such as flue gas (CO_2_/N_2_), and biogas (CO_2_/CH_4_) mixtures, where only CO_2_ molecules can overcome the structural transition energy barrier through specific interactions with the framework (e.g., hydrogen bonding of polar groups, metal sites, pore-confined van der Waals forces), triggering a cooperative change of the framework from cp to op [39–41]. Impurity gases cannot be adsorbed because of their weak molecular polarity and low interaction energy, thereby enabling efficient CO_2_ separation within the target pressure range.

Based on previous research, we systematically review the core mechanism of CO_2_ adsorption by flexible MOFs and the key strategies for flexible regulation. Focus on the structural dynamic transformation mechanisms driven by host–guest interactions, and analyze these phenomena with the typical case study of flexible MOFs for CO_2_ adsorption. Regarding flexible modulation, we will discuss how to control the structure–function relationship between framework flexibility and CO_2_ adsorption performance through metal node regulation, ligand design, and functionalization. Additionally, the application advantages and existing challenges of flexible MOFs in VPSA were discussed. We further summarized the key challenges faced by flexible MOFs in humidity condition, as well as in shaping. The strategies for functional integration and the preparation and application of composite adsorbents for CO_2_ adsorption processes were explored. The critical role of in situ characterization in elucidating the dynamic behavior of flexible MOFs during adsorption was emphasized. The possibility of using machine learning to assist in the design of flexible MOFs and predict CO_2_ adsorption performance was discussed.

## Mechanisms for CO_2_ Adsorption of Flexible MOFs

**Fig. 2:**
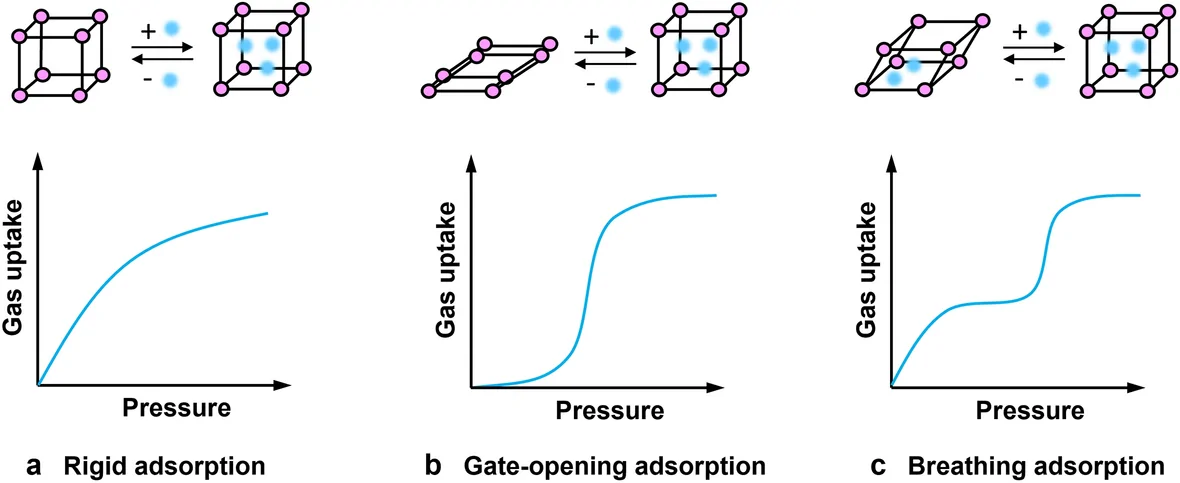
Schematic representation of **a** typical rigid, **b** gate-opening, and **c** breathing adsorption behaviors

The adsorption mechanisms of flexible MOFs for CO_2_ constitute the foundation that differentiates these materials from rigid porous adsorbents. Generally, the flexibility is intimately connected with special host–guest interactions. They possess a dynamic structural transformability that is sensitive to external stimuli. This structural flexibility is not merely a physical deformation but a thermodynamic transition between distinct metastable states, driven by the delicate interplay between the framework’s elastic strain and the host–guest interaction energy [35, 42, 43]. The unique behaviors allow for high working capacities and energy-efficient regeneration of flexible MOFs, which are expected to provide a modulable pore environment for CO_2_ capture. To comprehensively analyze this unique adsorption behavior, this section will first elucidate typical flexible behavior characteristics include the breathing effect and gate-opening effect (Fig. 2). The mechanisms detailed in this section are interrelated, linking framework changes to CO_2_ adsorption performance (e.g., capacity, selectivity, kinetics).

### Flexible Behaviors

#### Breathing Effect and Stepwise Adsorption

Some flexible MOFs exhibit “breathing” behavior during CO_2_ adsorption, which refers to the reversible phase transition of flexible MOFs under external stimuli (e.g., gas molecules, CO_2_ partial pressure, and temperature), involving at least the synergistic structural reconstruction between two phases (cp to op) and accompanied by notable alterations in cell parameters and volume. Its essence is the reversible transformation between different phases under thermodynamic conditions, which can be manifested as a clear shift in diffraction peak positions or a phase transition in in-situ X-ray diffraction (XRD), and the adsorption isotherm exhibits step-like features and hysteresis, etc.

**Fig. 3:**
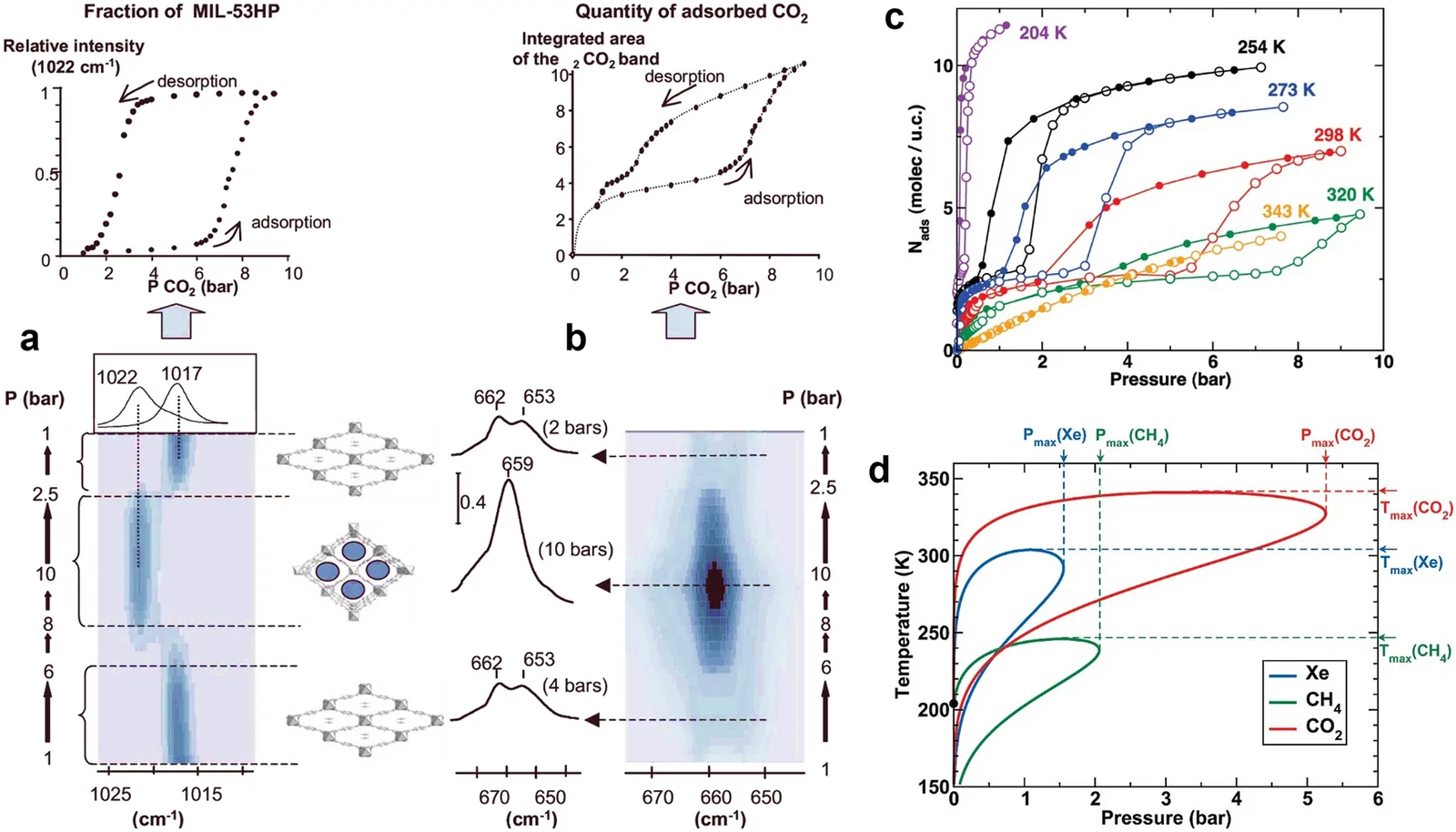
IR of (**a**) Variation in the intensities of MIL-53LP and MIL-53HP bands and (**b**) in the *v*_*2*_ CO_2_ bands versus CO_2_ pressure. Top: a sorption cycle (corresponding IR spectra). Reproduced with permission from Ref. [52], Copyright 2007, Wiley–VCH. **c** CO_2_ isotherms of MIL-53(Al). **d** Schematic representation of the adsorption stress for np (red) and lp (blue). Reproduced with permission from Ref. [46]. Copyright 2010, American Chemical Society. (Color figure online)

The most typical representative of respiratory flexible MOFs is the MIL-53 series [M(bdc)(OH)]n (bdc = 1,4-benzenedicarboxylate, M = Al, Cr, Sc, Fe, Ga, In) [44–49]. The framework is constructed through the interconnection of infinite trans chains of corner-sharing AlO_4_(OH)_2_ octahedra (linked via OH groups), mediated by BDC ligands [47]. The ligands connected to metal atoms can undergo distortion when adsorbing guest molecules, and as the pore structure opens, the adsorption curve presents a stepped form. The framework exhibits a reversible phase transition, accompanied by the switching of pore size from narrow (np, pore size ~ 0.8 nm) to large pores (lp, pore size ~ 1.4 nm) upon CO_2_ adsorption. The variation is caused by a modification in the carboxylic acid groups’ coordination mode [50]. The change in the metal-O-ligand angle drives the rhombus channel from “closed” to “open”. CO_2_ stabilizes the lp phase by interacting with the carboxylic acid oxygen or open metal site (OMS), resulting in a single/double step isotherm. Adsorbed CO_2_ molecules form strong guest-guest interactions along the channels (with intermolecular distances as short as 3.4 Å), while simultaneously establishing robust electron donor–acceptor (EDA) interactions between CO_2_ and the framework hydroxyl sites. The energy released from both interactions fully compensates for the deformation energy barrier required for the framework to undergo significant structural changes from the large-pore phase to the narrow-pore phase, driving the total system energy down to a stable range. The density functional theory (DFT) calculations performed on the loaded HT material indicated that CO_2_ molecules interacted directly with the hydroxyl groups. Gérard Férey and his team have conducted extensive research on the breathing phenomenon and phase transition mechanism of MIL-53 and its family during gas adsorption. By comparing the differences between MIL-53(Cr^3+^/Al^3+^) and MIL-53(V^4+^) during CO_2_ adsorption, it was found that the breathing effect of flexible MOFs is directly related to the interaction between gas molecule polarity (quadrupole moment) and framework-specific functional groups (–OH) [51]. In MIL-53(Cr^3+^/Al^3+^), the specific interaction between CO_2_ and *μ*_*2*_-OH groups drives framework contraction–expansion, whereas nonpolar CH_4_ cannot trigger this process, exhibiting conventional micropore filling adsorption behavior; MIL-47 (V^4+^) loses its specific interaction with CO_2_ due to the replacement of *μ*_*2*_-OH by *μ*_*2*_*-*O, resulting in the absence of a breathing effect in the framework. It exhibits conventional micropore adsorption for both CO_2_ and CH_4_. Furthermore, they revealed, for the first time through in situ experiments and characterization, how the interaction between CO_2_ and the MIL-53(Cr) framework drives structural expansion and contraction (Fig. 3c). During repeated pressure cycling (1–10 bar), rapid switching between low- and high-pressure states occurs without significant decay of the diffraction peaks, demonstrating the excellent cyclic stability of the breathing effect [46, 52, 53].

Additionally, a class of flexible MOFs typically features dual-interwoven three-dimensional channels, also known as interpenetrating networks, whose dynamic adsorption behavior is often described as breathing. For instance, [Zn_2_(BPnDC)_2_(bpy)]_n_ (BPnDC, benzophenone 4,4′-dicarboxylic acid, bpy, 4,4′-bipyridine) [54, 55], also known as SNU-9, the framework exhibits two-step adsorption behavior for both CO_2_ and H_2_, accompanied by significant desorption hysteresis, whereas it shows three-step adsorption for N_2_ and O_2_. Interestingly, the interlayer spacing of certain interpenetrating flexible MOFs also depends on the guest molecules, such as [Cu(dhbc)_2_bpy]·H_2_O, where Hdhbc = 2,5-dihydroxybenzoic acid and bpy = 4,4′-bipyridyl [56]. Other flexible MOFs exhibiting breathing effects include DUT-128(Ni) [57], MOF-508 [58], MIL-88 [59], BMOF-1-dcppy [60], and others.

**Fig. 4:**
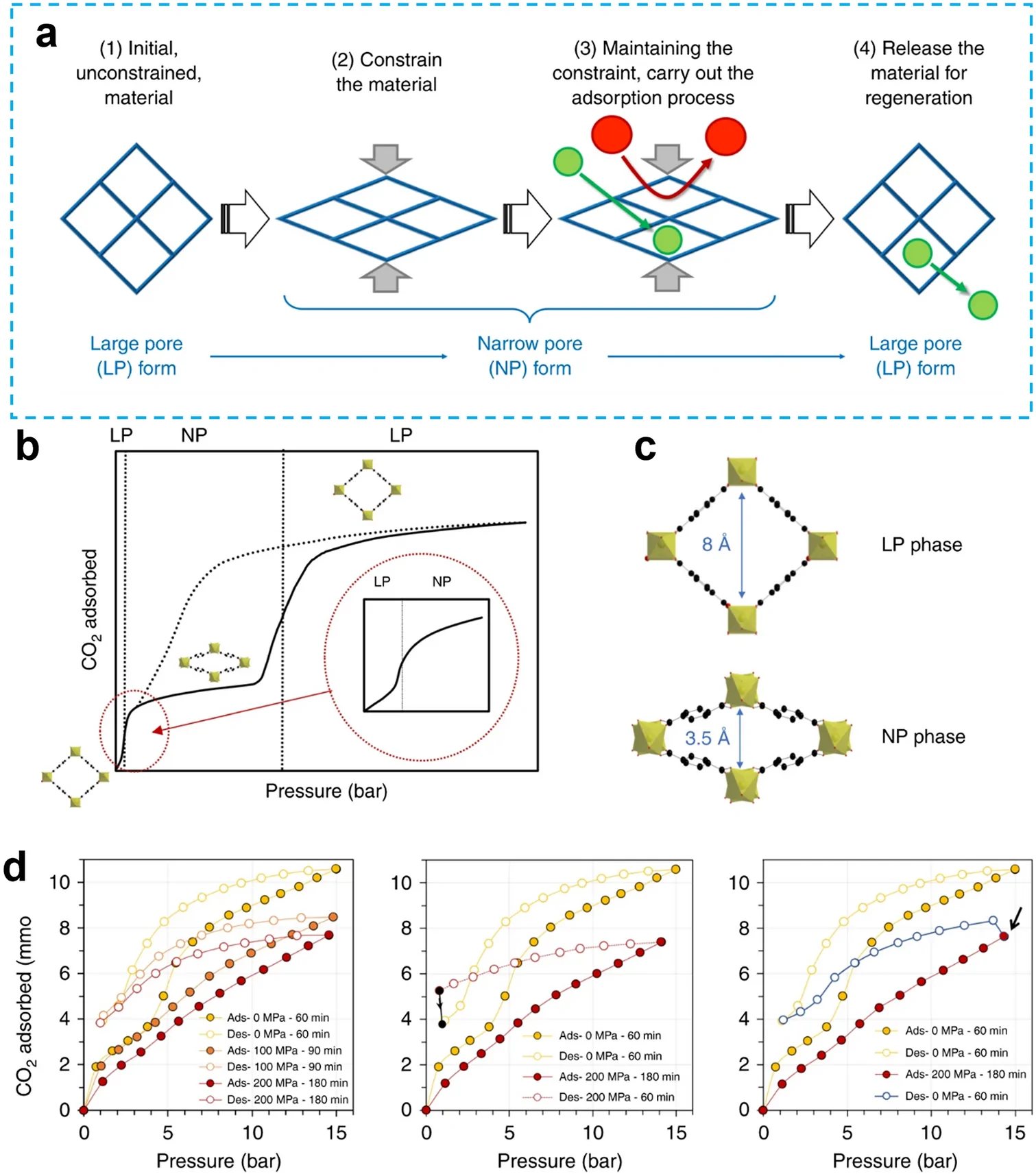
**a** Breathing behavior of MIL-53. **b** CO_2_ isotherm with a two-step process. **c** lp and np phases of MIL-53. **d** CO_2_ adsorption–desorption isotherms of MIL-53(Al) at 303 K. Reproduced with permission from Ref. [50]. Copyright 2020, The Authors

Notably, flexible MOFs with breathing effects can achieve efficient separation of mixed gases due to their two-step adsorption isotherms and pore-size variation. Chanut et al. focused on MIL-53(Al) to propose an innovative strategy for precisely regulating pore size through external mechanical pressure, enabling molecular sieve separation of gases with similar dimensions (CO_2_/N_2_, CO_2_/CH_4_) [50]. The core mechanism involves mechanically induced reversible structural transitions in MIL-53 (Al) coupled with pore size matching. Under mechanical pressure, a reversible structural change occurs in MIL-53 (Al) between a large-pore state (pore size 8 Å) and a narrow-pore state (pore size 3.5 Å) (Fig. 4a). Thus, the essence of selective adsorption lies in the precise matching of pore size to the kinetic diameter of the gas. Since the kinetic diameter of CO_2_ (3.3 Å) is smaller than the np phase pore size (3.5 Å), CO_2_ molecules can readily access the channels and achieve stable adsorption through interactions with framework hydroxyl groups. In contrast, the kinetic diameters of N_2_ (3.7 Å) and CH_4_ (3.8 Å) are both larger than 3.5 Å, preventing them from entering the np phase channels (Fig. 4b). Therefore, when mechanical pressure exceeds 200 MPa (MIL-53 fully transitions to the np phase), adsorption capacity approaches zero, and selectivity toward CO_2_ approaches infinity.

#### Gate Opening Adsorption

The “gate opening” effect is one of the most representative structural response behaviors of flexible MOFs. Initially, the pores may be closed or narrow (inaccessible to gas molecules). As the gas partial pressure increases, the MOF framework undergoes a structural change, and the pores “open”, allowing a large number of guest molecules (such as CO_2_) to enter and adsorb [19, 61]. This phenomenon is usually reflected in the “mutation” or “step” on the adsorption isotherm. At a particular critical pressure, the adsorption amount exhibits an abrupt increase. The adsorption kinetics process of the gate effect goes through four stages, including (i) initial stage: closed phase; (ii) induction stage: molecular triggering; (iii) structural change: open pore; (iv) adsorption equilibrium: open phase. This effect in flexible MOFs not only significantly enhances both the adsorption capacity and selectivity of MOFs for target gases (CO_2_) [62–65], but also provides a new strategy and theoretical basis for efficient gas separation [66–68].

**Fig. 5:**
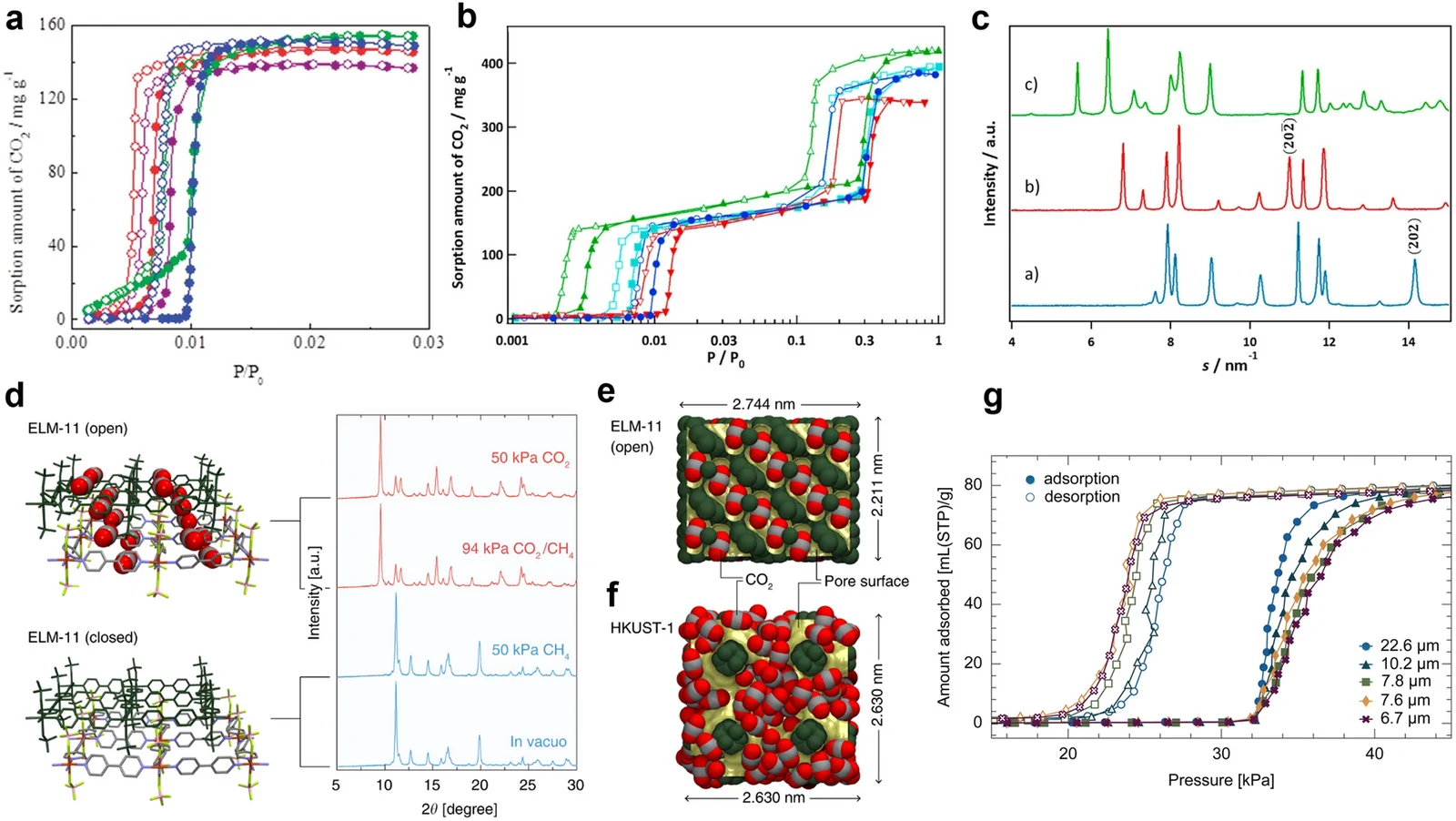
**a** Sorption isotherms for CO_2_ of pre-ELM-11 and pre-ELM-11 treated by different conditions. Reproduced with permission from Ref. [71]. Copyright 2011, American Chemical Society. **b** CO_2_ sorption isotherms of ELM-11 at different temperatures. **c** XRD patterns of ELM-11 at 195 K. Reproduced with permission from Ref. [72]. Copyright 2016, American Chemical Society. **d** In situ PXRD and crystal structures of ELM-11 in cp and op states. **e** and **f** Pore environment and CO_2_ configuration of ELM-11 and HKUST-1. Reproduced with permission from Ref. [73]. Copyright 2020, The Authors. **g** CO_2_ adsorption isotherms at 273 K of ELM-11 with various particle sizes. Reproduced with permission from Ref. [77]. Copyright 2024, The Authors

Kaneko and his co-workers studied several MOFs forming a similar two-dimensional network and their stacking structures. The authors termed these materials elastic layer-structured metal organic frameworks (ELMs) [69, 70]. One typical ELM exhibits a gate phenomenon, named [Cu(bpy)_2_(BF_4_)_2_]n (bpy = 4,4′-bipyridine), also known as ELM-11. This MOF shows gate-opening sorption isotherms of N_2_, Ar, CO_2_, and CH_4_. They discovered that CO_2_ adsorption increased the interlayer spacing of the material from 0.458 to 0.578 nm, a 26% expansion. This dynamic structural transformation constitutes a novel reversible expansion/contraction regulation of two-dimensional layered stacked compounds induced by gas adsorption. The interaction between the pore walls and gas molecules gives rise to the gate-opening phenomenon. Subsequently, in their 2011 report, they discovered that different pre-adsorption pretreatment methods could alter the gate pressure of ELM-11 [71]. The ethanol-treated sample (e-ELM-11-vac) showed significantly reduced gating pressure during CO_2_ adsorption. Ethanol molecules weakened interlayer attractions through weak interactions with Cu centers or BF_4_^−^, such as hydrogen bonding and dipole interactions, thereby lowering the energy barrier for CO_2_ induced structural transitions (Fig. 5a). The Δ*H* of the clathrate for e-ELM-11-vac and ELM-11 were measured as 23.7 and 25.1 kJ mol^−1^, respectively, indicating a lower enthalpy change for the former. So, the CO_2_ solid interaction could also become weaker after ethanol treatment. The interlayer expansion induced by CO_2_ loading is ultimately governed by the balance between CO_2_–interlayer and interlayer–interlayer interactions. Once the weakening of interlayer interactions becomes predominant, a lower amount of CO_2_ adsorption is sufficient to induce cooperative structural transitions, which manifests as lower gate-opening pressures in the adsorption isotherms. This mechanism offers a novel strategy for regulating the adsorption behavior of flexible MOFs, enabling optimization of their gas separation performance through the design of solvent molecule polarity and size. Subsequently, in a 2016 paper by Kanoh and colleagues, the two-step gating phenomenon of ELM-11 in CO_2_ adsorption was investigated (Fig. 5b). Through experiments and simulations, the structural transformation mechanism and thermodynamic properties were elucidated [72]. XRD analysis confirms that both adsorption steps involve stepwise expansion of the layered structure, with the second step driving a monoclinic-to-triclinic transition and altering the interlayer stacking arrangement (Fig. 5c). Furthermore, simulation calculations indicate that CO_2_ preferentially occupies fixed sites during the first adsorption step, while the second step fills the expanded interlayer spaces, forming a stable guest-framework interaction network.

In 2020, Shotaro Hiraide demonstrated in their paper that ELM-11 exhibits pressure-dependent rapid response capabilities in the gated adsorption process of CO_2_, while also possessing the core advantage of adapting to rapid PVSA [73]. The structural transformation rate of ELM-11 was investigated through time-resolved in situ synchrotron XRPD measurements (Fig. 5d). Under an adsorption pressure of 40.8 kPa CO_2_ at 273 K, the structural transition of ELM-11 from cp to op can be completed within 10 s; when the CO_2_ pressure is increased to 250 kPa (at 298 K), 95% of the structural transition can be achieved in just 1.5 s. When pressure decreases at a rate of 2.4 kPa s^−1^, the phase transition from op to cp can be completed within 5 s. This is attributed to the “squeeze desorption” effect of framework contraction on CO_2_, resulting in significantly faster desorption kinetics than those of traditional rigid adsorbents such as HKUST-1 (Fig. 5e, f). This research establishes a foundation for the industrial application of flexible MOFs in VPSA. In subsequent reports, they combined molecular dynamics (MD) simulations with crystal structure analysis to reveal the dual permeation pathways of CO_2_ during the ELM-11 gating process and the “interlayer cooperative deformation” mechanism, specifically “interlayer horizontal permeation” and “interlayer stacking direction permeation” [74, 75]. This mechanism demonstrates that gating kinetics in MOFs can be optimized by modulating interlayer flexibility, via ligand length adjustment or modification of the metal center coordination environment. It also provides a clear structural optimization direction for designing highly efficient CO_2_ separation materials, such as VPSA adsorbents. The hysteresis loops commonly observed during the adsorption–desorption process of flexible MOFs increase energy consumption during regeneration. The hysteresis in the stepped isotherms of some flexible MOFs arises from the energy penalty associated with the growing cp—op interfacial area during the cp to op transition. The reverse op to cp transition does not need to surmount this interfacial energy barrier, thus the desorption branch of the isotherm matches the thermodynamic equilibrium, whereas the adsorption branch reflects the system trapped in a metastable state [32, 42, 76]. Furthermore, the phenomenon where the width of these hysteresis loops increases as particle size decreases remains poorly understood in terms of its underlying mechanism. Homare Arima et al. used a layered flexible MOF (ELM-11) as a model system, through experimental characterization (SEM, XRD, CO_2_ adsorption isotherms) and multiscale simulations, including nanoscale unit-cell molecular simulations and mesoscale Ising lattice models. They revealed the particle-size-dependent mechanism by which guests (CO_2_) induce structural phase transitions (Fig. 5g) [75, 77]. It supplies a theoretical support for improving the separation efficiency of flexible MOFs through particle size regulation.

When the pressure rose from 0 to 100 kPa, the benzimidazolate ligands in zeolitic imidazolate framework ZIF-7 (Zn(PhIm)_2_, PhIm = benzimidazolate) also rotated to open up cavities for CO_2_ adsorption [20, 78, 79]. It belongs to the SOD topological structure along with ZIF-8 (methylimidazole ligand). Zn^2+^ binds to the 1, 3-position N atoms in the ligand in a tetrahedral coordination manner, forming a rigid skeleton similar to zeolite, but it has a flexible response ability because of the ligand’s motion. The gate-opening pressure of ZIF-7 toward CO_2_ was determined to be 60 kPa at 303 K [80]. Notably, the CO_2_ adsorption–desorption of ZIF-7 shows a distinct hysteresis loop: the desorption gate closure pressure (approximately 40 kPa) is lower than the opening pressure. This phenomenon arises from the bistable nature of ZIF-7, in which the wide-pore and narrow-pore phases coexist under identical thermodynamic conditions, with phase transition requiring the surmounting of a specific energy barrier. Furthermore, temperature influences the phase transition tendency of ZIF-7, thereby regulating its gating behavior. Within the range of 273–323 K, ZIF-7 is more likely to maintain the wide-pore phase at lower temperatures (e.g., 273 K), exhibiting higher CO_2_ adsorption capacity. When the temperature rises above 308 K, the thermodynamic equilibrium of the system shifts toward the narrow-pore phase, resulting in a notable reduction in the capacity for adsorption. At 303 K, ZIF-7 preferentially forms a wide pore phase during heating, while maintaining a narrow pore phase during cooling, reflecting the “memory effect” of phase transition. Therefore, CO_2_ absorption may be significantly impacted by slight variations in temperature or pressure. The author revealed the crystal structure characteristics of the wide pore phase of ZIF-7 through structural analysis. The benzimidazole ligand tilts toward the sodalite cage’s center, and the cavity inside the cage is filled with CO_2_ molecules. The rotational motion of the ligand is the core driving force for the transition from np to lp. Electrostatic interactions and van der Waals forces between CO_2_ and ligands provide rotational energy for the ligands, breaking the structural constraints of the np phase and enabling pore expansion. The adsorption isotherm of ZIF-7 for CH_4_ also exhibits a gate opening phenomenon; however, compared to CO_2_, the gate opening pressure is higher [81]. Due to the lack of a quadrupole moment and weak interaction with ligands, CH_4_ requires higher pressure to accumulate sufficient adsorption capacity to drive ligand rotation. The high quadrupole moment of CO_2_ generates a strong electrostatic interaction with the N atom of the benzimidazole ligand, providing energy for ligand rotation and reducing the phase transition energy barrier. This also constitutes the core reason why the gate pressure of CO₂ is considerably lower than that of CH_4_.

**Fig. 6:**
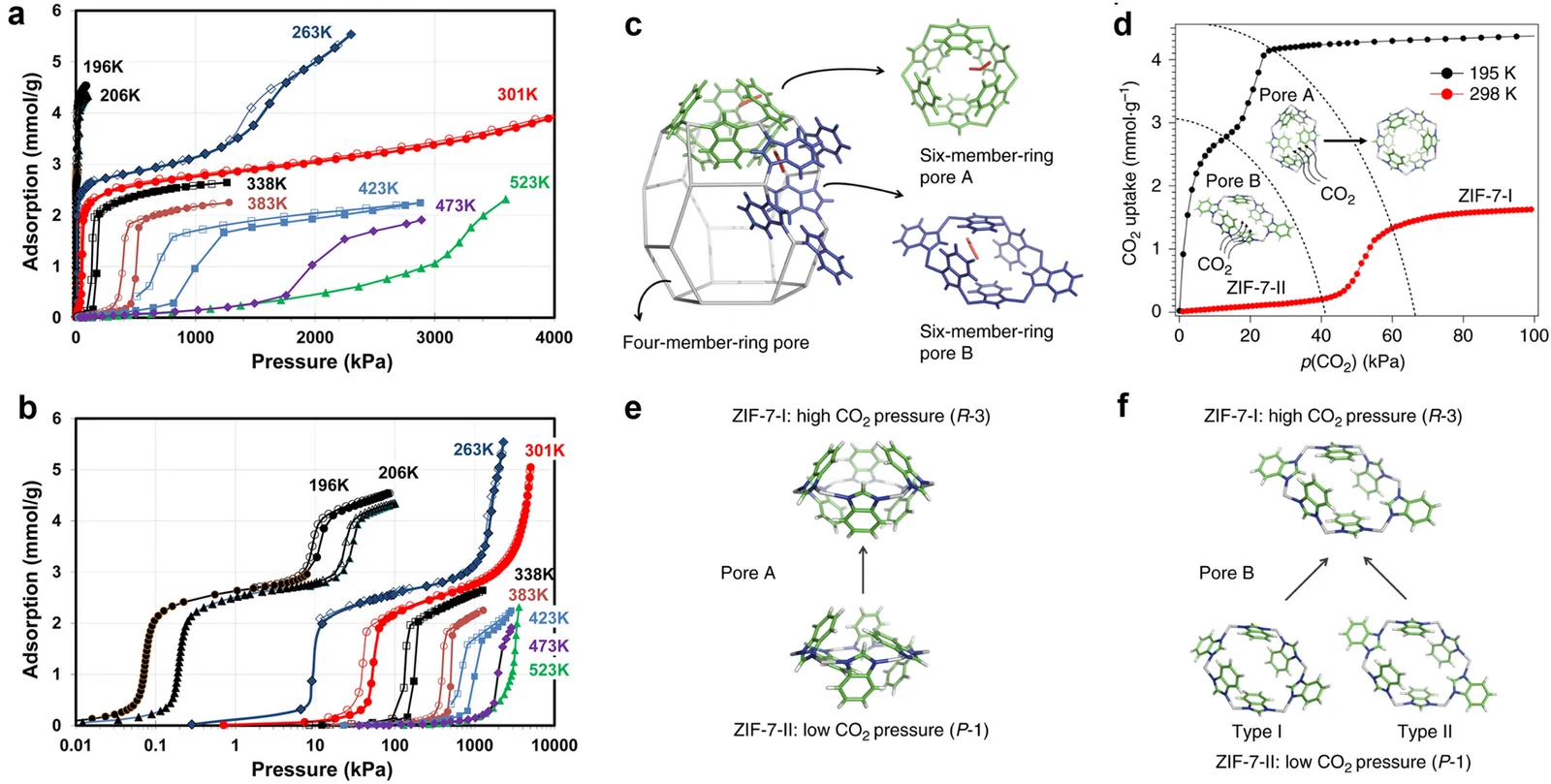
CO_2_ adsorption behavior and structures of ZIF-7. **a** and **b** CO_2_ adsorption and desorption isotherms of ZIF-7. Reproduced with permission from Ref. [82]. Copyright 2015, American Chemical Society. **c** Sodalite cage of ZIF-7 with two types. **d** CO_2_ isotherms at 195 K and 298 K. **e** and **f** The structural change of pore A and pore B. Reproduced with permission from Ref. [84]. Copyright 2019, The Authors

Overall, the complex phase transition behavior of ZIF-7 during CO_2_ adsorption requires analysis from both thermodynamic and kinetic perspectives. In thermodynamics, the phase transition of ZIF-7 adsorbing CO_2_ is categorized into two distinct phases, each corresponding to different temperature ranges, with fundamentally different thermodynamic driving mechanisms: (i) low temperature phase transition (196–383 K) is a CO_2_-induced transition from np to lp. The phase transition characteristic of this stage is the “double step” phenomenon of adsorption isotherms, whose thermodynamic essence is enthalpy-dominated host–guest interactions. (ii) High-temperature phase transition (460–700 K) is an entropy-driven np to lp transformation (Fig. 6a, b). This description is characterized as a “spontaneous phase transition” in the absence of an external agent, with its thermodynamic essence being a decrease in free energy driven by entropy increase. Otherwise, dynamics, the phase transition of ZIF-7-II to ZIF-7-I is not a simple expansion of a single pore channel, but rather a continuous kinetic process involving “initial adsorption–ligand rotation–inter-pore migration–structural rearrangement” of CO_2_ within non-uniform pores [82, 83]. Zhao et al. explained in their article that the core structural feature of ZIF-7 is non-uniform porosity. There are four types of pore channels: two hexagonal ring pores A/B (Fig. 6c), one quaternary ring pore, and a cubic sodium gabion. Among them, the six membered ring pore A (traditionally considered as an “active open pore”) is connected to pore B (adjacent six membered ring pores) through a shared benzimidazole ligand. Before phase transition, the CO_2_ adsorption energy in pore B is only 6.9 kJ mol^−1^ higher than that in the A-B pore channel. CO_2_ initially accumulates in pore B and can migrate through the channel upon thermal activation. After the phase transition, the CO_2_ adsorption energy at site B is 14.1 kJ mol^−1^ higher than at site A, causing CO_2_ to preferentially fill pore B before entering pore A. DFT calculations indicate that the phase transition energy difference Δ*E*_*f*_ between the two phases of ZIF-7 is approximately 29.9 kJ mol^−1^ per unit cell. The adsorption affinity of CO_2_ can overcome this phase transition energy barrier. This structure provides a unique channel for CO_2_ migration and is also the structural basis for phase transition kinetics (Fig. 6d) [84, 85].

The Kitagawa group reported a series of coordination polymers with an interdigitated structure (CID) family that also shows a gate-opening phenomenon for CO_2_. They designed CID-3{[Zn(ndc)(bpy)]}_n_ (CID-3; ndc = 2,7-naphthalene dicarboxylate) with nonpolar pore walls and an interdigitated structure [86]. Its pore parameters and structural responsiveness are adapted to CO_2_ capture requirements, to show high CO_2_ selectivity in a ternary mixture of CO_2_, N_2_, and O_2_. Subsequently, two structurally similar yet ligand-substituted flexible MOFs featuring an interdigitation motif of 2D layers were synthesized: CID-5 ({Zn(5-NO_2_-ip)(bpy)}_n_, 5-NO_2_-ip = 5-nitroisophthalate, bpy = 4,4′-bipyridyl) and CID-6 ({Zn(5-MeO-ip)(bpy)}_n_, 5-MeO-ip = 5-methoxyisophthalate) [87, 88]. The two compounds display entirely different gate opening behaviors for CO_2_ and CH_4_, with the fundamental difference stemming from the electronic and steric effects of the ligand substituents. The strong electron-withdrawing character of 5-NO_2_-ip induces severe structural contraction of the framework after desorption; in contrast, the electron-donating character of 5-MeO-ip stabilizes the framework and suppresses significant structural transformation. However, the pure-phase MOF exhibited poor selectivity. Subsequently, a ligand-based solid solution (CID-5/6G) of CID-5 and CID-6 was innovatively constructed to regulate gate-opening behavior, achieving higher CO_2_/CH_4_ selectivity. Moreover, a Cu-based 3D flexible MOF Cu(FMA)(4,4′-Bpe)_0.5_ (FMA = fumarate; 4,4′-Bpe = trans-bis-(4-pyridyl)ethylene)[89] with double interpenetration, also showed a gate-opening adsorption toward CO_2_, thus resulting in significant adsorption selectivities for CO_2_/CH_4_ and CO_2_/N_2_ separations.

**Fig. 7:**
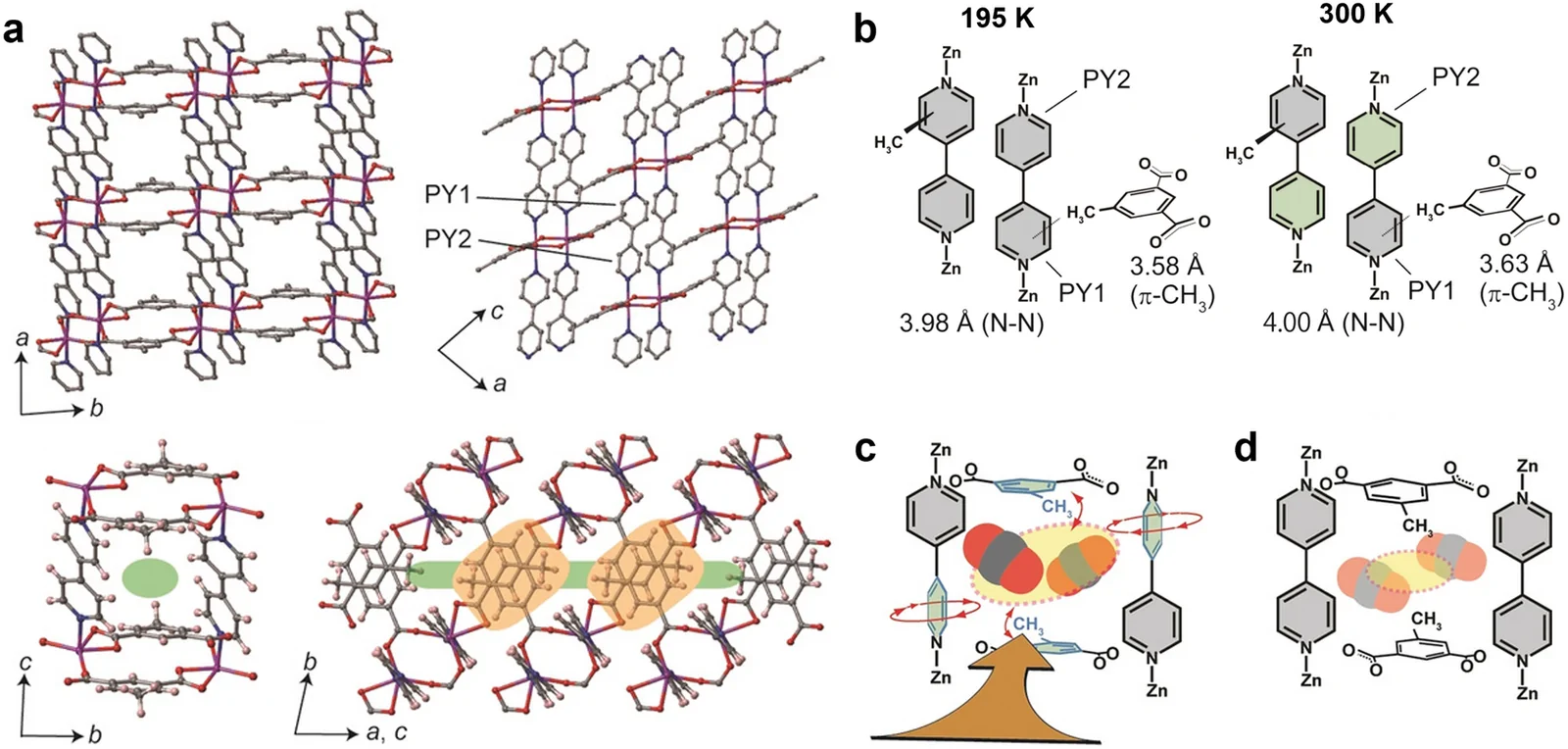
**a** 2D coordination network of CID-Me at 195 K. **b** Pyridyl rings with local environments. **c** and** d** CO_2_ adsorption through the rotational rotors. Reproduced with permission from Ref. [90]. Copyright 2018, Wiley–VCH

The adsorption characteristics of conventional porous materials typically show higher adsorption uptake at lower temperature, yet some materials exhibit an unusual phenomenon of high adsorption at high temperature. Few studies have explored the influence of temperature on gating effects, such as CID-Me([Zn(5-Meip)(bpy)]n, 5-Me-ip = 5-methylisophthalate) [90]. Sharma et al. investigated the influence of temperature on the gating effect and simulated the CO_2_ diffusion process in CID-Me through multiscale calculations [91] (Fig. 7). By examining structural motion, diffusion efficiency, and energy barriers, they revealed that the increased CO_2_ adsorption amount at elevated temperatures is due to thermal activation, which decreases the kinetic constraints on gate opening. This provides theoretical guidance for designing porous adsorbents characterized by high adsorption capacity at elevated temperatures. Recently, Amombo Noa et al. synthesized a novel chiral lanthanide flexible MOF (CTH-17), which shows a pressure-triggered gating effect in CO_2_ adsorption [92]. Its flexibility stems from the full-framework synergy between cpb6- ligand motion and the elongation of LaO_6_ rod-like SBUs. It also demonstrates high adsorption capacity at higher temperatures. However, the authors did not further discuss the cause of this unusual phenomenon.

**Fig. 8:**
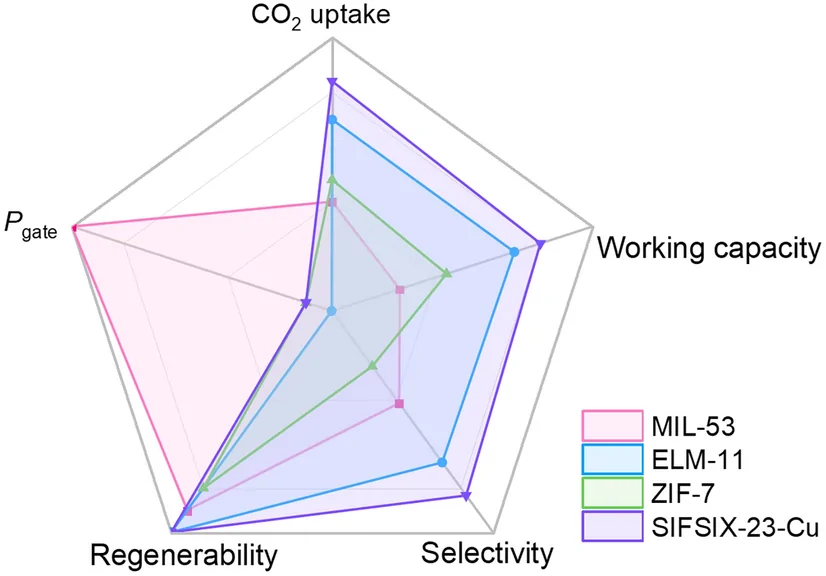
Semi-quantitative Radar chart for the adsorption performance of several representative flexible MOFs (*P*_gate_: gate-opening pressure, CO_2_ uptake: mmol∙g.^−1^, selectivity: CO_2_/N_2_)

Consequently, the strength of host–guest interactions (including hydrogen bonding, van der Waals forces, *π*-*π* stacking, and electrostatic interactions) directly determines the critical gate-opening pressure, the magnitude of adsorption enthalpy change, and the reversibility of phase transition during the adsorption process. The framework deformation energy is a key thermodynamic parameter governing the phase transition behavior of flexible MOFs. It is defined as the energy difference between cp and op in the absence of guest molecules, with no guest-guest or host–guest interactions considered [35, 93]. Depending on the deformation energy, the phase transition pressure of the system can occur over different pressure ranges (Table 1 and Fig. 8). Some systems exhibit multistep phase transitions, indicating the existence of an intermediate phase (ip phase) between the op and cp states, with a pore volume intermediate between the two. From a thermodynamic perspective, when the interaction energy derived from guest–host and guest–guest interactions is enough to offset the framework deformation energy, pore opening of the framework is triggered, which gives rise to the characteristic S-shaped adsorption isotherm. Thus, the modulation of deformation energy provides a critical guide for designing flexible MOFs [94].

**Table 1:** Comparison of structural transition parameters for representative flexible MOFs

Flexible MOFs	Transition type	Guest molecule	Temp. (K)	*P*_gate_ (bar)	Δ*H* (kJ∙mol^−1^)	References
MIL-53	Breathing	CO_2_	304	> 5	32	[51, 52]
ELM-11	Gate-opening	CO_2_	273	0.01	25	[71]
ZIF-7	Gate-opening	CO_2_	298	0.4–0.5	30	[84]
CID-5/6	Gate-opening	CO_2_	195	0.01–0.02	–	[87]
SIFSIX-23-Cu	Gate-opening	CO_2_	298	0.5	45–51	[95]

### Molecular Recognition and Size Selectivity

During carbon capture processes, it is often necessary to selectively capture target CO_2_ molecule from multicomponent mixtures. However, traditional materials, such as rigid MOFs and zeolites, struggle to simultaneously address the dual challenges. Flexible MOFs can achieve molecular recognition through object-induced structural transformations (gating effects). However, current systems often suffer from preferential adsorption of high-affinity molecules and co-adsorption of non-target molecules after gating, lacking the ability to selectively recognize target molecules. It requires the adsorbent to possess three key characteristics. Firstly, controllable framework flexibility is the key to achieving kinetic gating selectivity. Kinetic gating is a stimulus-responsive adsorption behavior dominated by guest mass transfer kinetics. The flexible frameworks can tune the dynamic aperture size and diffusion channel tortuosity via their local or global flexibility, to amplify the diffusion rate difference between guest molecules with similar physicochemical properties [96]. Additionally, the material’s structure features precisely matched pore size (CO_2_, 3.3 Å), which can enhance separation selectivity. Furthermore, binding sites within the pores must interact with the target molecule CO_2_, with synergistic enhancement of recognition capability between sites. Therefore, achieving multi-mechanism synergy enables CO_2_ exclusive recognition even when confronted with competitively similar molecules of comparable size and higher affinity [94, 97–100].

**Fig. 9:**
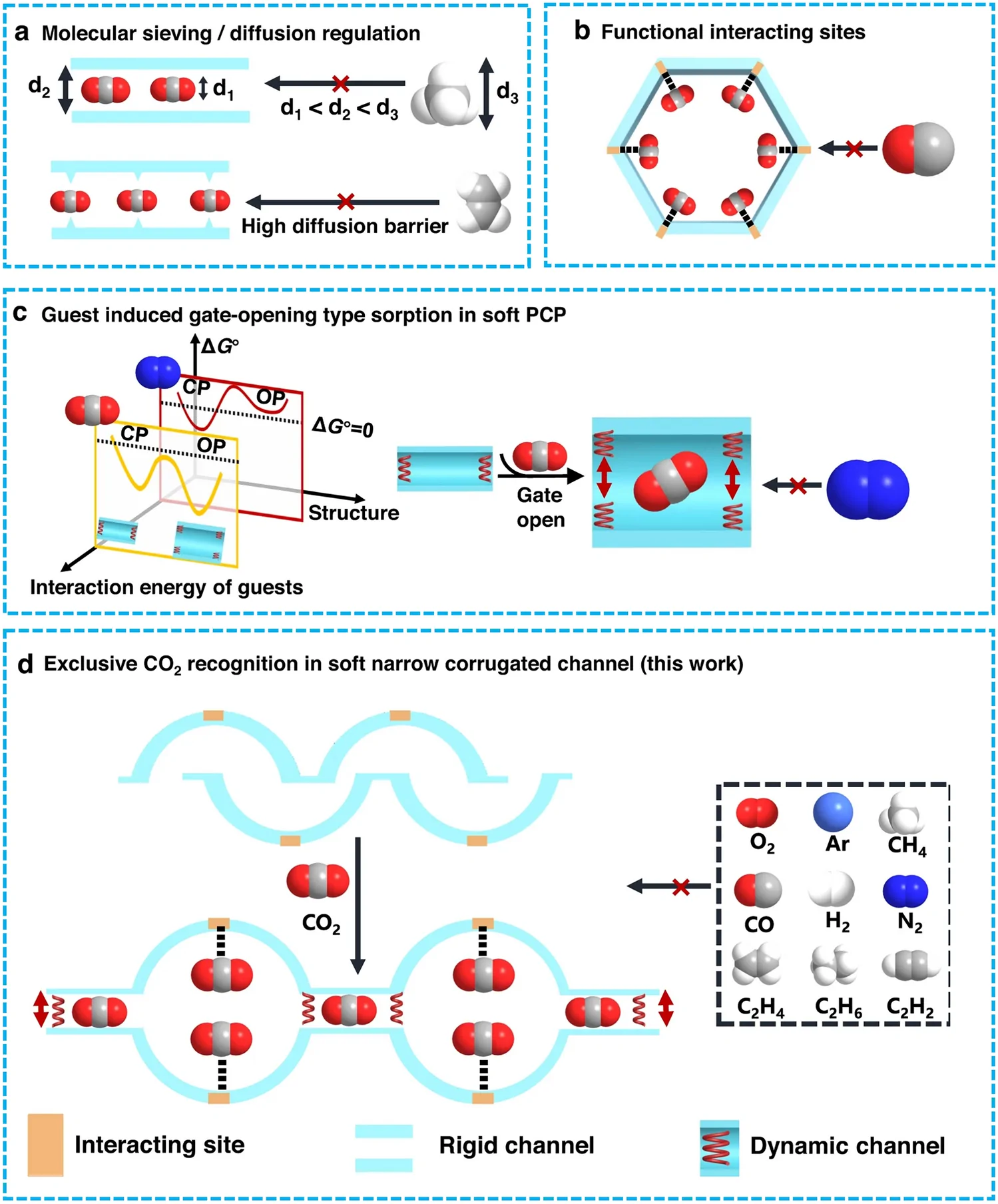
Four types of exclusive molecular recognition mechanisms. **a** Size-exclusion or diffusion-limited effect. **b** Functional interacting sites. **c** Interaction energy surpassed structural deformation energy triggers from the cp phase to the op phase transformation and guest adsorption. **d** Exclusive discrimination gating (EDG). Reproduced with permission from Ref. [102]. Copyright 2023, The Authors

Research on the type of flexible MOFs for the recognition of small molecules is limited. As early as 2010, Chen et al. reviewed how to design MOFs with small molecule recognition [101]. They proposed three functional MOF design strategies, including: (i) precise control of the pores in the original cubic network MOF to achieve size exclusion separation, such as Cu(FMA)(Pyz)_0.5_; (ii) by introducing functional units such as open metal sites and Lewis acid–base sites into MOF pores, the specific interactions with small molecules can be enhanced, such as Cu_2_(BPTC)(H_2_O)_2_·(DMF)_3_(H_2_O), HKUST-1, MOF-505; (iii) microporous mixed-metal–organic frameworks (M’MOFs) for small molecule identification and kinetic molecular sieving separation, such as Zn_3_(BDC)_3_[Cu(Pyen)]·(DMF)_5_(H_2_O)_5_ (M’MOF 1). Recently, Gu et al. designed a flexible MOF [Co(3,5-pdc)dpg]_n_ (PCP-3,5-pdc), which stands out as an exceptional representative of molecular recognition [102]. It is a flexible MOF featuring narrow corrugated channels that achieves an “exclusive discrimination gating (EDG)” effect for CO_2_ by synergistically regulating the channel’s three-dimensional structure, binding site distribution, and framework flexibility. It can selectively adsorb CO_2_ from among 10 similar gas molecules (Fig. 9), including CH_4_, N_2_, O_2_, H_2_, CO, C_2_H_2_, C_2_H_4_, Ar, and C_2_H_6_. During activation, PCP-3,5-pdc transforms from its synthetic state (phase α) into an activated state (phase *β*) with an interdigitated 2-D layer structure. Guest-free PCP-3,5-pdc initiates a structural transformation into another phase (phase *γ*) when the CO_2_ relative pressure (*P*/*P*_0_) reaches 0.05. Such a structural change is associated with the sudden pore opening, transitioning from a thick pore (phase *β*) to a wide-pore configuration (phase *γ*) upon CO_2_ adsorption. They reveal two types of CO_2_ binding sites: site I in the broader cavity and site II in the narrow window, which enhance selectivity through differential interactions. Although C_2_H_2_ exhibits higher binding energy, it must overcome greater framework deformation energy and thus fails to trigger gating. In consequence, by regulating the three-dimensional structure of the channel and the flexibility of the framework, it is possible to overcome the performance limitations of conventional separation materials and achieve efficient capture of target molecules in complex mixed gases.

### Other Guest-Induced Structural Transformation

As the foundational driving mechanism underpinning the flexible behaviors and selective CO_2_ binding of flexible MOFs, guest-induced structural transformation exhibits distinct mechanistic features and regulatory rules that are tightly coupled to the phase state of the CO_2_-containing guest system. The materials show reversible structural change or phase transition during solvent exchange or gas adsorption/desorption processes [103, 104]. In gaseous guest systems, cumulative CO_2_-framework interactions trigger stepwise structural transitions (breathing, gate-opening) at threshold partial pressures, consistent with the macroscopic behaviors described in Sect. 2.1. Weak interactions between the framework and the guest molecules stabilize the loaded state, leading to the framework becoming thermodynamically favored. Hence, when the guest molecules enter or exit the channel, the coordination unit will be slightly displaced in a direction that benefits interaction with the guest. This phenomenon can be observed in both the gas and liquid phases. More importantly, guest-induced structural transformation during CO_2_ adsorption requires special consideration of the alignment between CO_2_ molecules and the pore environment [41]. Because precisely engineering the adsorbent pore size to match the kinetic diameter of CO_2_ is extremely challenging.

**Fig. 10:**
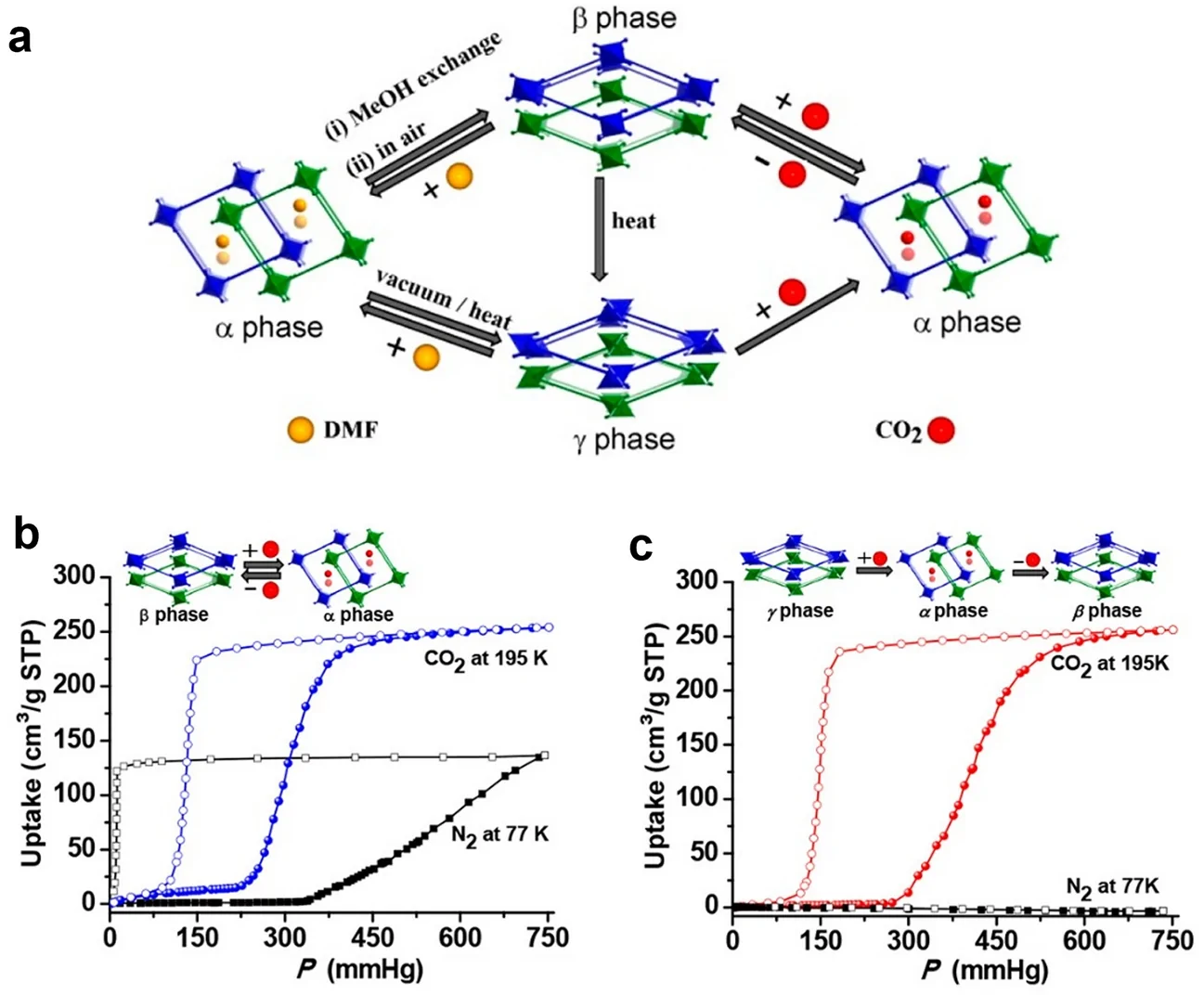
**a** Guest-induced structural changes of X-pcu-5-Zn. **b** and **c** CO_2_ (195 K) and N_2_ (77 K) sorption isotherms of X-pcu-5-Zn-β and X-pcu-5-Zn-γ. Reproduced with permission from Ref. [105]. Copyright 2018, American Chemical Society

**Fig. 11:**
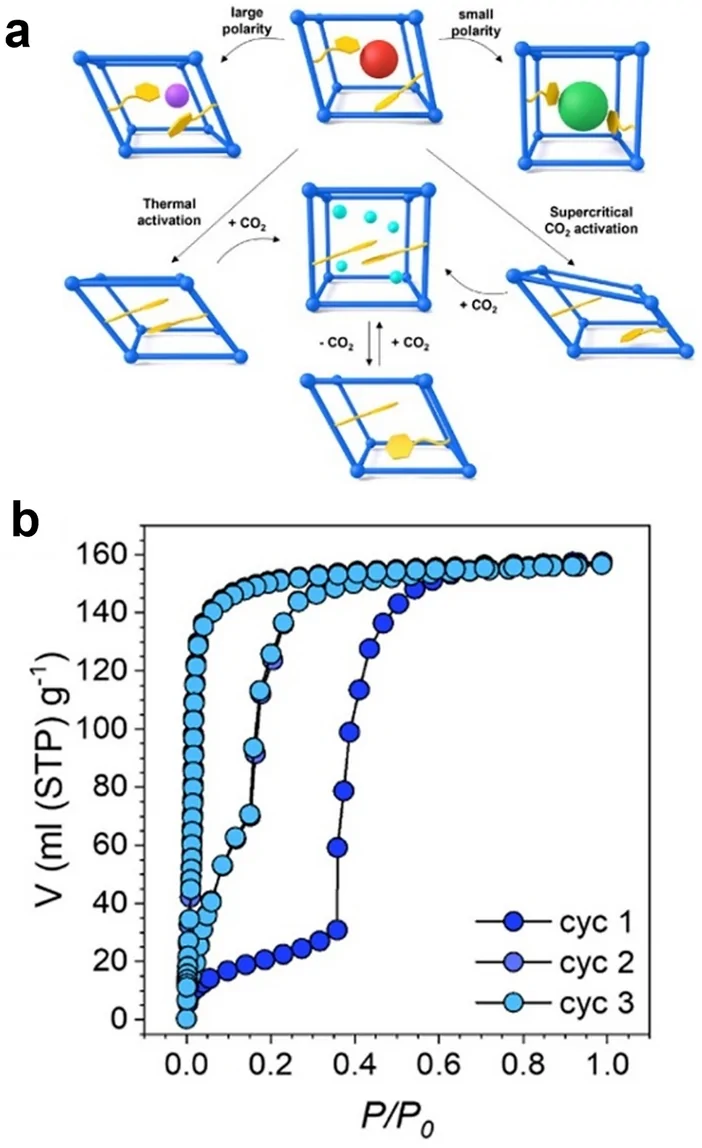
**a** Phases change reversibly induced by different external triggers. **b** CO_2_ sorption isotherms of PCP-1-cp-TH. Reproduced with permission from Ref. [107]. Copyright 2024, The Authors

Typically, CO_2_-induced dynamic changes in pore size were also observed in ZIF-7. The movement of CO_2_ molecules between two types of pores achieves a two-step adsorption of the dynamic framework structure, which is the breathing effect mentioned earlier. The large window and low adsorption energy of pore B make it the initial adsorption site, and the dynamic opening of pore A depends on the accumulation of CO_2_ in pore B. Theoretical calculations and structural analysis have demonstrated that the migration of CO_2_ in the non-uniform pores of ZIF-7 affects the dynamic changes in the structure [84]. In addition, Zhu et al. reported a flexible, X-pcu-5-Zn, with three crystal forms, two of which are non-porous and homomorphic. Here, the bond rearrangement triggered by CO_2_ molecules leads to the sliding of interpenetrating network structures, rotational deformation of organic linkers, or changes in the orientations of coordination bonds [105]. Furthermore, the interaction between the host and guest can also complete specific adaptation based on the adsorbate-induced framework. After 1 day of methanol exchange and vacuum desorption of the guest, the *α*-phase (porous) undergoes a single-crystal-to-single-crystal (SCSC) transformation to form the *β*-phase. The *β*/*γ* phase (non-porous) can be transformed into the α phase by opening pores through different pathways under DMF immersion or gas molecule (CO_2_) induction (Fig. 10). NKU-FlexMOF-1 is also a flexible microporous MOF with adaptive structural properties and adjustable host–guest interactions, whose function varies with changes in sorbate and temperature [106]. Recently, Wang et al. reported a new flexible PCP-1 ([Zn_2_(bndc)_2_(bpy)]_n_) that uses intraframework π–π interactions to combine both local and global flexibility, or pendant group motion [107]. The guest-induced structural transformation of PCP-1 is essentially the regulation of *π*–*π* stacking rearrangement by guest molecules through energy input. Solvent molecules induce the switching of three open pore phases through polarity or size differences, and the activation mode forms two initial closed pore phases through energy differences. CO_2_ adsorption triggers a cyclic transition from open to new closed phases (Fig. 11). This multiphase transformation ability enables PCP-1 to switch the gate pressure of CO_2_ adsorption by regulating the guest type or activation mode.

**Fig. 12:**
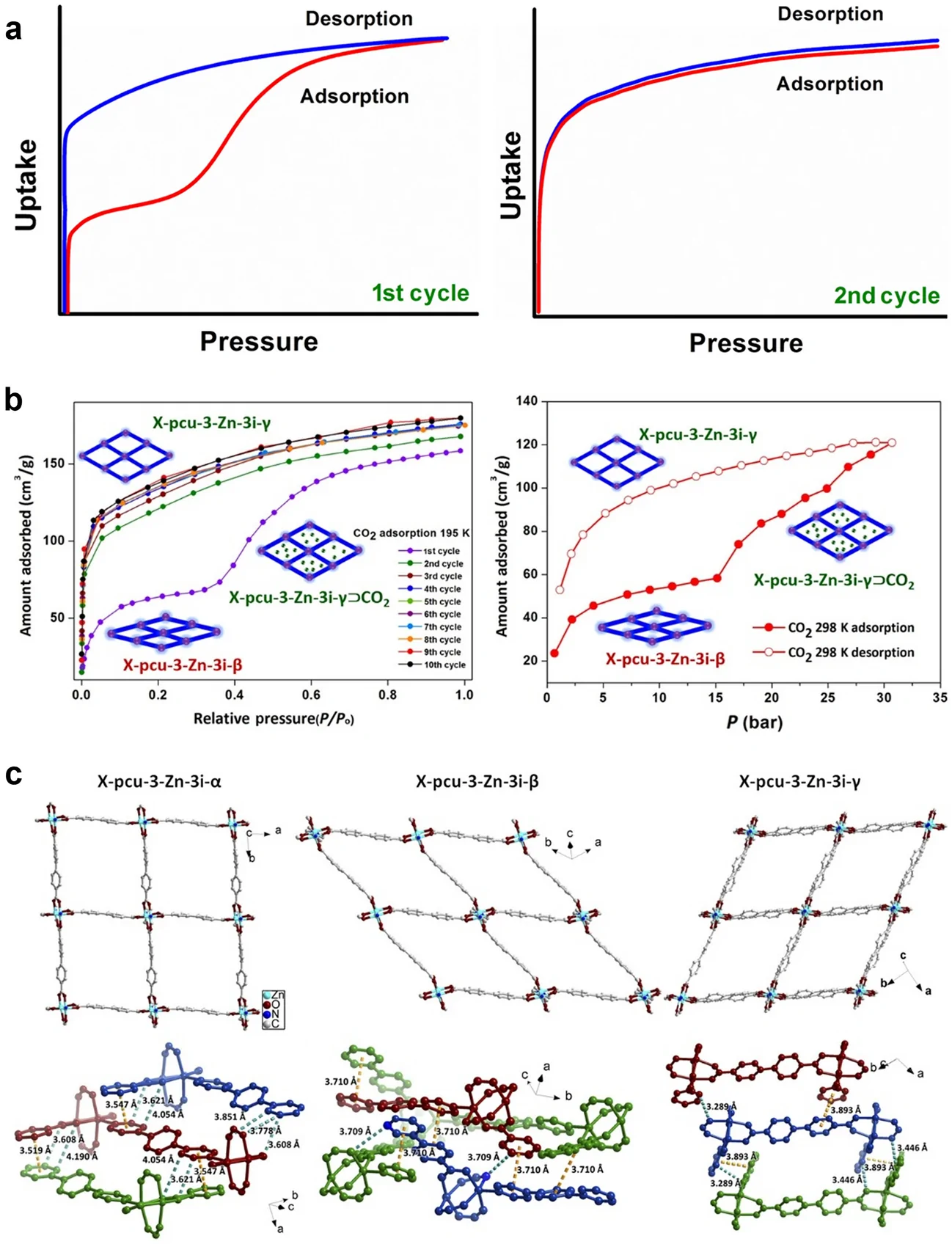
**a** Typical isotherms of flexible MOFs with a shape-memory effect. **b** CO_2_ sorption of X-pcu-3-Zn (left: 195 K, low pressure; right: 298 K, high pressure). **c** *sql* networks of the *α*, *β*, and *γ* forms of X-pcu-3-Zn-3i. Reproduced with permission from Ref. [111]. Copyright 2018, The Authors. This publication is licensed under CC BY-NC

Additionally, the shape-memory effect in flexible MOFs involves a guest-induced structural transformation [108]. The adsorption characteristic of the shape memory effect enables a transition from flexible to rigid during adsorption. Sakata et al. reported [Cu_2_(bdc)_2_(bpy)]_n_ with a dual interpenetrating framework, whose crystal size reduction modulates the structural flexibility of the coordination framework and triggers a shape memory effect [109]. When the crystal size is reduced to the mesoscopic scale, an unusual metastable open-dried phase is also formed. The reduction in crystal size suppressed structural mobility, stabilized the open dry phase, and successfully separated two convertible empty phases (the closed phase and the open dry phase). Therefore, the material has switchable adsorption properties and can exhibit either gate-opening or non-gate-opening behavior. Later, Hong et al. also observed this dynamic gate-opening phenomenon during N_2_ and Ar adsorption, and it disappeared during the second adsorption cycle [110]. X-pcu-3-Zn-3i represents another porous material with a shape-memory effect, and it is the first one where this effect is induced by multiple sorbates [111]. Shape memory effects can be observed under several gas molecule stimuli (CO_2_ at 195 K, N_2_ at 77 K, CO at 82 K, and high-pressure CO_2_ at 298 K) (Fig. 12). The triple interpenetrating topology is the core structural foundation of its dynamic behavior—interpenetrating networks have no chemical bonds between them and can undergo structural reconstruction through relative sliding. At the same time, mutual restraint among multiple networks prevents framework collapse during the transformation process, balancing flexibility and stability. Subsequently, Yang et al. reported CPM-107, which has an anionic framework encapsulating ordered extra-framework cations and solvent molecules. It achieved, for the first time, the unique combination of a lock-and-key effect (i.e., guest-selective and irreversible gating) with shape-memory properties [112]. Its desolvation-induced closed phase (CPM-107-cp) is inert to common gases such as N_2_ and H_2_, yet can be specifically “unlocked” by CO_2_ at 195 K, triggering a gated structural transformation to form a rigid porous phase (CPM-107-op). Recently, they reported another flexible SIFSIX coordination network, SIFSIX-23-Cu^N^ [95]. Through single-atom ligand modification, the benzene ring ligand (L = 1,4-bis(1-imidazolyl)benzene) of the parent material SIFSIX-23-Cu was replaced with a pyridine ring ligand (L^N^ = 2,5-bis(1-imidazolyl)pyridine), resulting in significant shape memory properties. The discovery of shape-memory effects provides new insights into the development of novel intelligent porous adsorbents.

## Flexible Regulation

**Fig. 13:**
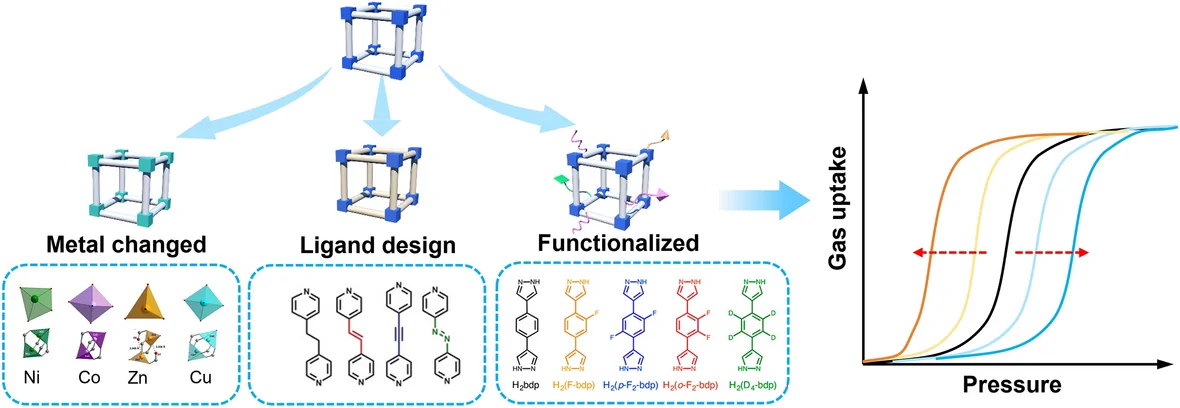
Strategies for flexible regulation. Reproduced with permission from Ref. [117]. Copyright 2023, The Authors. Reproduced with permission from Ref. [118]. Copyright 2019, Wiley–VCH. Reproduced with permission from Ref. [119]. Copyright 2016. American Chemical Society

The dynamic behavior of flexible MOFs is governed by a complex interplay of factors. It is not solely dictated by the intrinsic mobility or binding affinity of the metal nodes and organic linkers (Fig. 13), but is also significantly driven by the guest-induced deformation of the macroscopic framework [113–116]. They are critical factors in controlling selective adsorption behavior, as they influence the energy state of framework deformation during adsorption/desorption processes. For adsorbents exhibiting type-F-IV isotherms, maximizing the working capacity relies heavily on aligning the gate-opening and gate-closing transitions with the specific operational pressures for gas loading and delivery. Consequently, the capacity to precisely modulate these phase-transition pressures in flexible MOFs is crucial for designing porous materials optimized for distinct gas adsorption and storage.

### Metal Centers

**Fig. 14:**
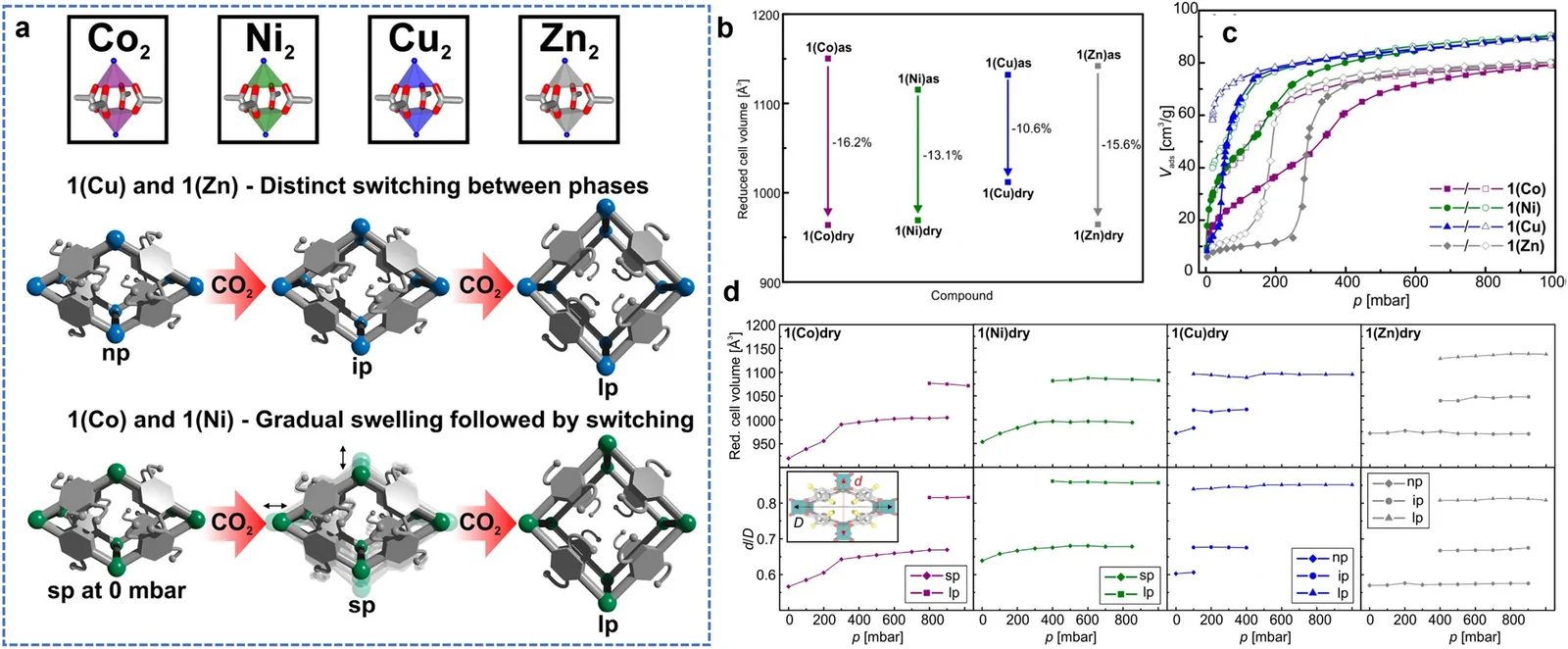
Flexibility difference of metal centers. **a** Schematic depiction of two different phase transitions for CO_2_ adsorption. **b** Cell volume changes from lp to np phase. **c** CO_2_ sorption isotherms at 195 K of 1(Co), 1(Cu), 1(Ni), and 1(Zn). **d** Evolution of the reduced cell volume and the *d*/*D* ratio derived from in situ PXRD data (acquired during CO_2_ adsorption at 195 K). Reproduced with permission from Ref. [124]. Copyright 2018, American Chemical Society

Owing to their distinctive structural features, the precise synthesis of flexible MOFs is crucial for realizing their distinctive dynamic response functions. Compared with conventional rigid MOFs, the synthesis of flexible MOFs requires more meticulous ligand design, careful selection of metal nodes, and stringent control of preparation conditions to ensure that the materials exhibit the desired dynamic behavior while maintaining structural stability [120]. Among them, the single- and mixed-metal MIL-53 are the most representative (M^3+^  = Al^3+^, Fe^3+^, Cr^3+^, In^3+^, …) [48, 121, 122]. Some metals do not trigger their flexible adsorption, and certain metal centers can block the flexible behavior. Klein et al. investigated the influence of metal ion substitution on the guest-induced flexibility of M_2_(ndc)_2_(dabco) (M^2+^  = Co^2+^, Cu^2+^, Ni^2+^, Zn^2+^; ndc = 2,6-naphthalenedicarboxylate; dabco = diazabicyclo[2.2.2]octane; DUT-8) [123]. DUT-8(M) exhibits obvious changes in gas adsorption performance and variation of flexibility. DUT-8(Ni) was previously found to exhibit breathing behavior, and its Co(II) analog also shows similar properties. This may be attributed to the specific electronic structures of different metal ions. In addition to noncovalent interactions between side-chain groups and the linker backbone, distortion of the coordination structure is another key factor that induces material flexibility. To further investigate this mechanism, the research team synthesized a series of pillared-layered M_2_(BME-bdc)_2_(dabco) MOFs (M^2+^  = Zn^2+^, Co^2+^, Ni^2+^, Cu^2+^; BME-bdc^2−^  = 2,5-bis(2-methoxyethoxy)-1,4-benzenedicarboxylate; dabco = diazabicyclo[2.2.2]octane) [124], thereby expanding the study of the transition between narrow-pore and macroporous phases. Interestingly, during solvent removal, the extent of pore contraction varies markedly with the metal node: the Co(II) material exhibits the largest contraction, whereas the Cu(II) material, with its relatively rigid coordination environment, undergoes the smallest structural change (Fig. 14). This phenomenon can be attributed to differences in the electronic properties of the metal ions when disrupting the square-pyramidal geometry required for the lp phase. In CO_2_ adsorption experiments, Zn‑ and Cu‑based MOFs display distinct adsorption gate-opening pressures, and three pore states are identified: narrow-pore, medium-pore, and large-pore. By contrast, the Ni(II) and Co(II) materials show a stepwise gas-filling process at higher pressures, followed by a complete structural transition to the lp phase.

### Ligand Design and Functionalization

The dynamic behavior of flexible MOFs largely depends on the design of the organic ligands [117, 125]. For example, in the synthesis of the MIL‑53 family, terephthalic acid (BDC) is employed as the ligand; its carboxylate groups can switch between monodentate and bidentate coordination modes under different pressures, thereby triggering the material’s breathing effect [126]. Similarly, when the coordination number at the metal center remains unchanged, the linker conformation has a substantial impact on framework flexibility—a conclusion demonstrated by studies of DUT‑8(Ni) [127, 128]. The conformational isomers of the 2,6‑ndc linker can modulate the framework’s flexibility. The length of the carboxylic acid linker can also be adjusted to control flexibility. For example, replacing 1,4-BDC with a longer 1,4-NDC and 9,10-ADC can be used to regulate the structural flexibility and C_2_H_2_/CO_2_ selectivity of Zn-MOFs [129].

Moreover, the addition of substituents to organic linkers (also known as functionalization) is believed to profoundly influence the dynamic properties of flexible MOFs [26, 114]. The core goals are to modify the pore environment or specific response characteristics of the material without compromising the MOF topology, crystallinity, or stability. This was confirmed in a systematic study of MIL-88 [Fe_3_O(L)_3_(H_2_O)_2_(OH)] (where L = 1,4-bpdc (88B), 4,4′-bpdc (88D)), by introducing functional groups onto the benzene ring to alter its swelling degree, such as CH_3_, CF_3_, NH_2_, NO_2_, OH, F, Cl, Br [130]. A larger functional-group size and a higher density of such groups within the interlayer space lead to a reduced breathing amplitude. In addition, by tuning host–guest interactions or diffusion energy barriers introduced by functionalization, the material’s swelling behavior in the liquid phase can be markedly enhanced, and “breathing” can even be induced in nonpolar solvents.

**Fig. 15:**
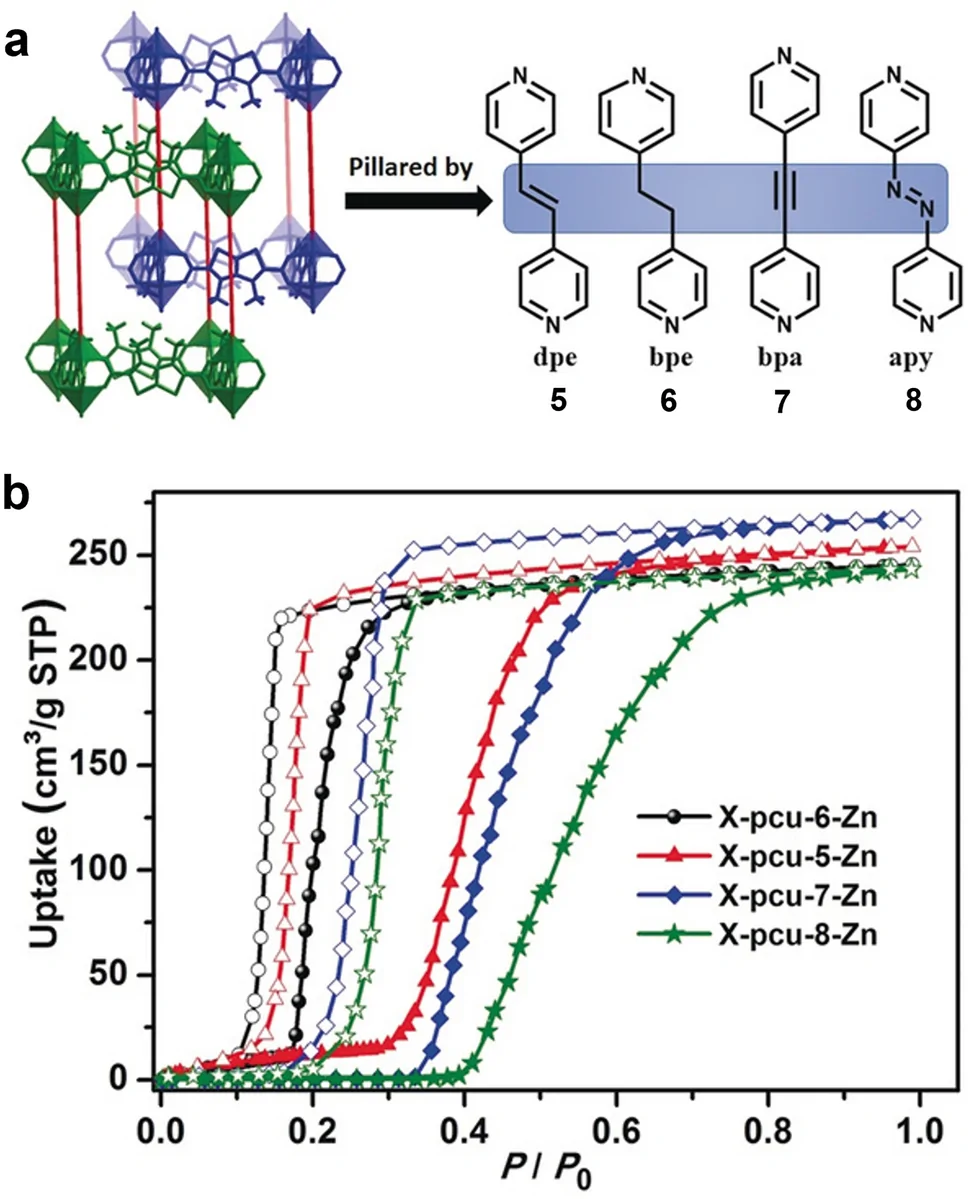
**a** 3D twofold-interpenetrated framework (X-pcu-n-Zn) was prepared with four pillar linker ligands. **b** CO_2_ sorption isotherms of X-pcu-n-Zn-β at 195 K. Reproduced with permission from Ref. [118]. Copyright 2019, Wily-VCH

When considering practical applications, the gate-opening or closing pressure of flexible MOFs must fall within the pressure range for CO_2_ storage and release. Research indicates that introducing ligands with conformational variability or rotational degrees of freedom can alter the gate pressure of flexible adsorbents. Zhu et al. conducted a study in the pcu-topology MOF, where they replaced dpe (1,2-di(4-pyridyl)ethylene) with bpe (1,2-bis(4-pyridyl)ethane), bpa (1,2-bis(4-pyridyl)acetylene), and apy (4,4'-azopyridine) through linker substitution (Fig. 15). This enabled rational control over the flexible adsorption behaviors of CO_2_, C_2_H_2_, and C_2_H_4_ [118]. Another representative example is MIP‑203‑F/S/M (F = fumaric acid; S = succinic acid; M = malic acid) [131]. Using three linkers of comparable molecular size, researchers achieved a transition from rigidity to flexibility in MOFs by exploiting differences in structural degrees of freedom and bond rotational mobility. A contrast in structural dynamics emerges between the rigid fumarate and the more flexible succinate. By comparison, MIP‑203‑M exhibits pronounced framework‑expansion flexibility along with suitable local bond distortions or bending toward guest molecules, delivering excellent performance in CO_2_/N_2_ adsorption and separation.

**Fig. 16:**
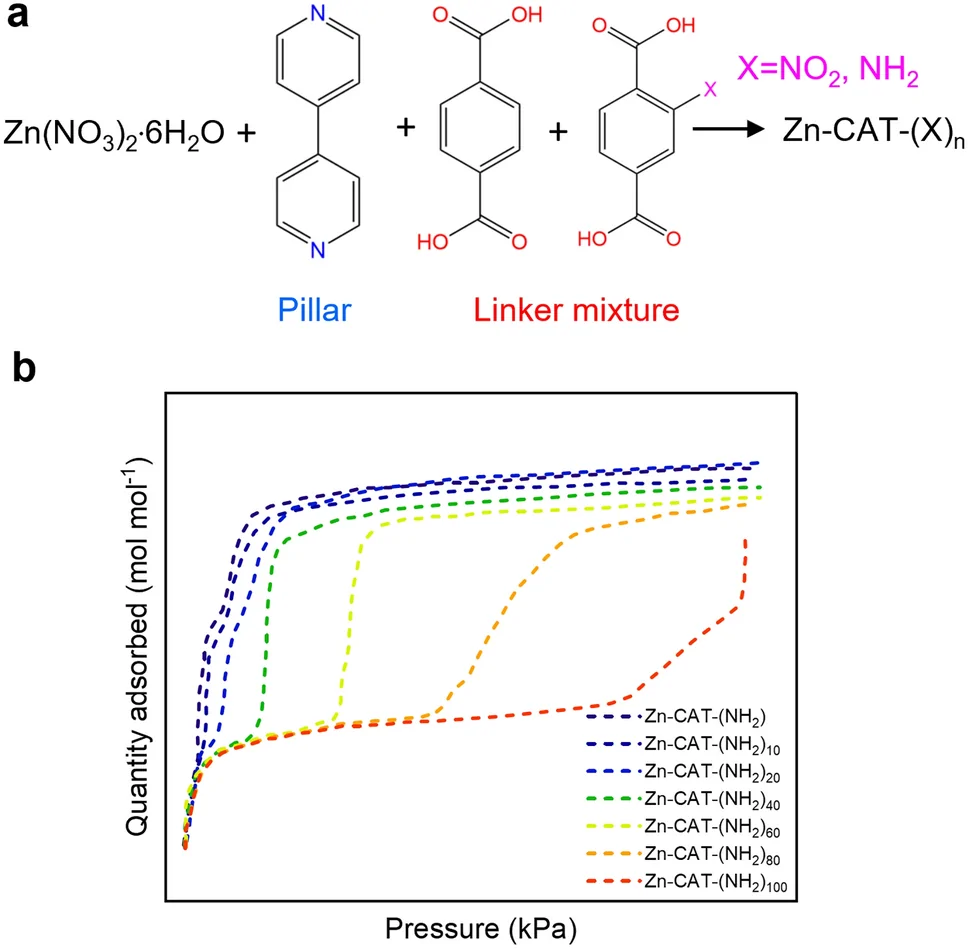
**a** Scheme of Zn-CAT-(X)n with various ratios of bdc-X linkers (X is –NO_2_ or –NH_2_). **b** Schematic of CO_2_ adsorption isotherms for all Zn-CAT-(NH_2_)n at 195 K. Reproduced with permission from Ref. [132]

The mixed linker strategy can also effectively regulate the pressure range of flexible adsorbents. Bonneau et al. employed a dual interpenetrating flexible MOF [Zn_2_(bdc)_2_(4,4′-bpy)] (Zn-CAT) as their research subject [132]. By introducing –NH_2_ or –NO_2_ groups onto the benzene of the bdc linker and adjusting the mixing ratio of linkers, they successfully regulated the gate-opening pressure of Zn-CAT (Fig. 16). The gate-opening pressure (*P*_go_) and gate-closing pressure *(P*_gc_) depend on the deformation energy required to create space, which originates from interactions between the two interpenetrated frameworks. However, during the opening stage, the dominant factor is the evolving affinity of the linker/substituent. Compared to bare Zn-CAT, introducing –NH_2_ increases the gate opening pressure. This occurs because the amino group forms hydrogen bonds with adjacent bpy and carboxylate ligands, thereby reducing the framework’s flexibility. Conversely, the –NO_2_ substituent lowers the opening pressure due to the repulsive interaction exerted between it and the carboxylate ligand under guest-free circumstances.

### Functionalized

The gating threshold pressure of conventional ZIF-7 is relatively high (≈50 kPa), and its CO_2_ adsorption selectivity remains to be optimized. Researchers have designed a novel flexible MOF through boron doping: boron-doped ZIF-7 (B-ZIF-7). This approach introduces the boron-bridged benzimidazolate (B(bim)_4_^−^) ligand into the ZIF-7 framework for the first time, constructing a cationic framework and enabling the incorporation of tunable anions (NO_3_^−^, Cl^−^, OTf^−^) [133]. The B-ZIF-7 (anion) structure forms a cationic framework similar to ELMs, allowing free selection of guest anions. This enables precise regulation of CO_2_ adsorption gate-opening behavior while enhancing CO_2_ adsorption capacity at low pressures. Meanwhile, Krista S. Walton et al. employed a postsynthetic ligand exchange (PSE) strategy to introduce nitrogen-functionalized ligands (2-aminobenzimidazole, benzotriazole, and 5-azabenzimidazole) into the ZIF-7 framework, enabling precise control over its gated-opening behavior [134]. This approach preserved the CO_2_-induced phase transition while significantly enhancing CO_2_/CH_4_ separation performance by increasing CO_2_ adsorption affinity and optimizing the gating threshold pressure and hysteresis loop width.

**Fig. 17:**
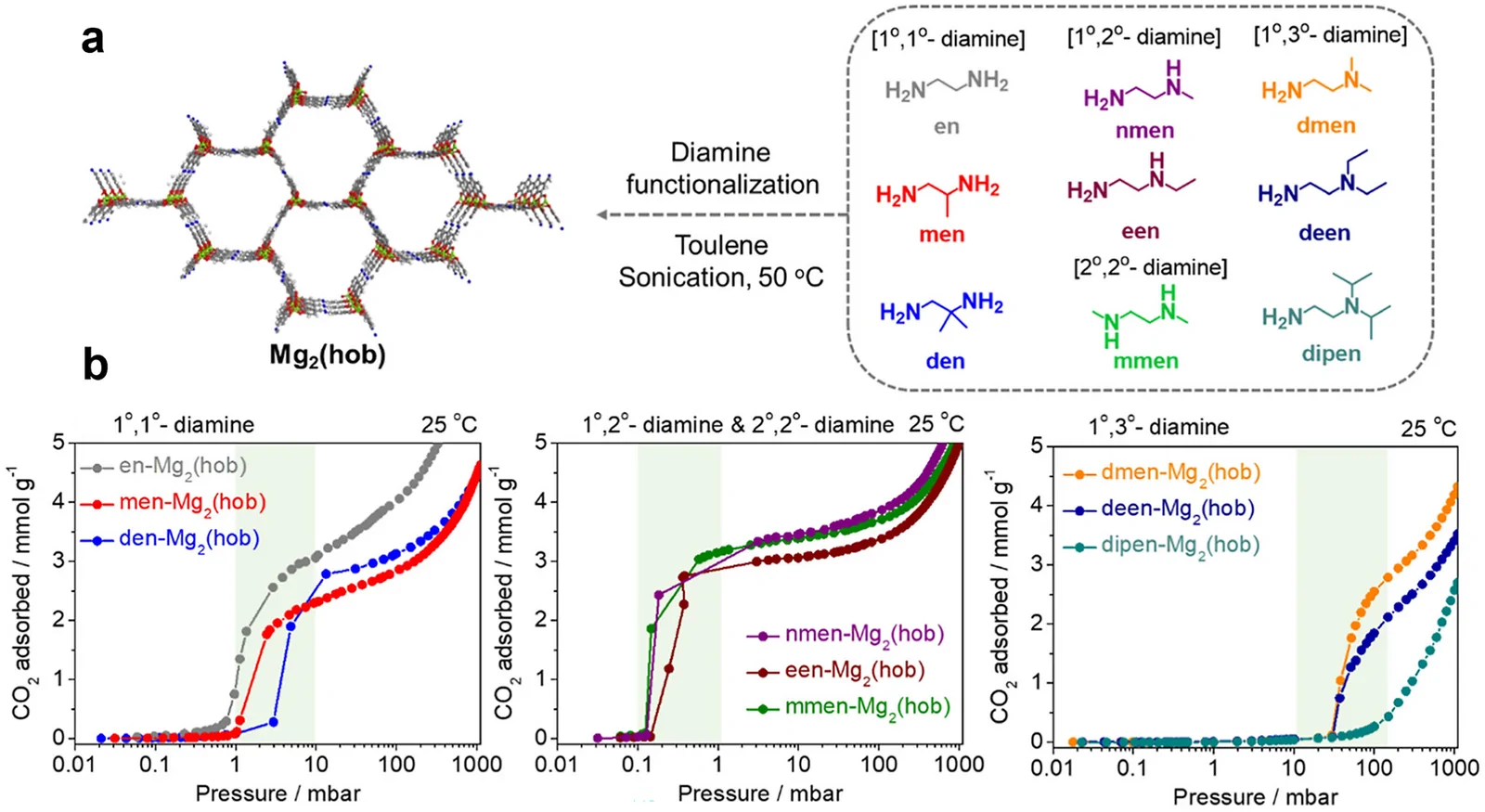
**a** Diamine functionalization of the MOF framework using various types of diamines. **b** CO_2_ adsorption isotherms at 298 K. Reproduced with permission from Ref. [138]. Copyright 2024, American Chemical Society

Furthermore, some of these amine-modified MOFs can function as “phase-change” adsorbents. Their CO_2_ adsorption isotherms not only exhibit a unique step- shape but also undergo significant shifts with temperature changes. Attaching alkyl diamines to unsaturated metal sites within specific MOF pores has been demonstrated to effectively enhance their capacity and selectivity for CO_2_ at low-pressure (Fig. 17) [135–139]. McDonald and his co-workers introduced mmen(4,4′-dioxobiphenyl-3,3’-dicarboxylate) into M_2_(dobpdc) (M = Mg, Mn, Fe, Co, Zn) to synthesize a phase-change adsorbent exhibiting a step-shaped CO_2_ adsorption isotherm, enabling efficient capture of flue gas CO_2_ [140]. Its mechanism involves CO_2_ molecules inserting into metal-amine bonds to form carbamate groups, while adjacent amine groups undergo proton transfer to form ammonium ions, ultimately assembling into ordered ammonium carbamate chains. This synergistic reaction requires reaching a metal-dependent threshold pressure to be triggered.

## Advantages and Potential Applications of Flexible MOFs

### High Working Capacity and Ideal Selectivity

**Fig. 18:**
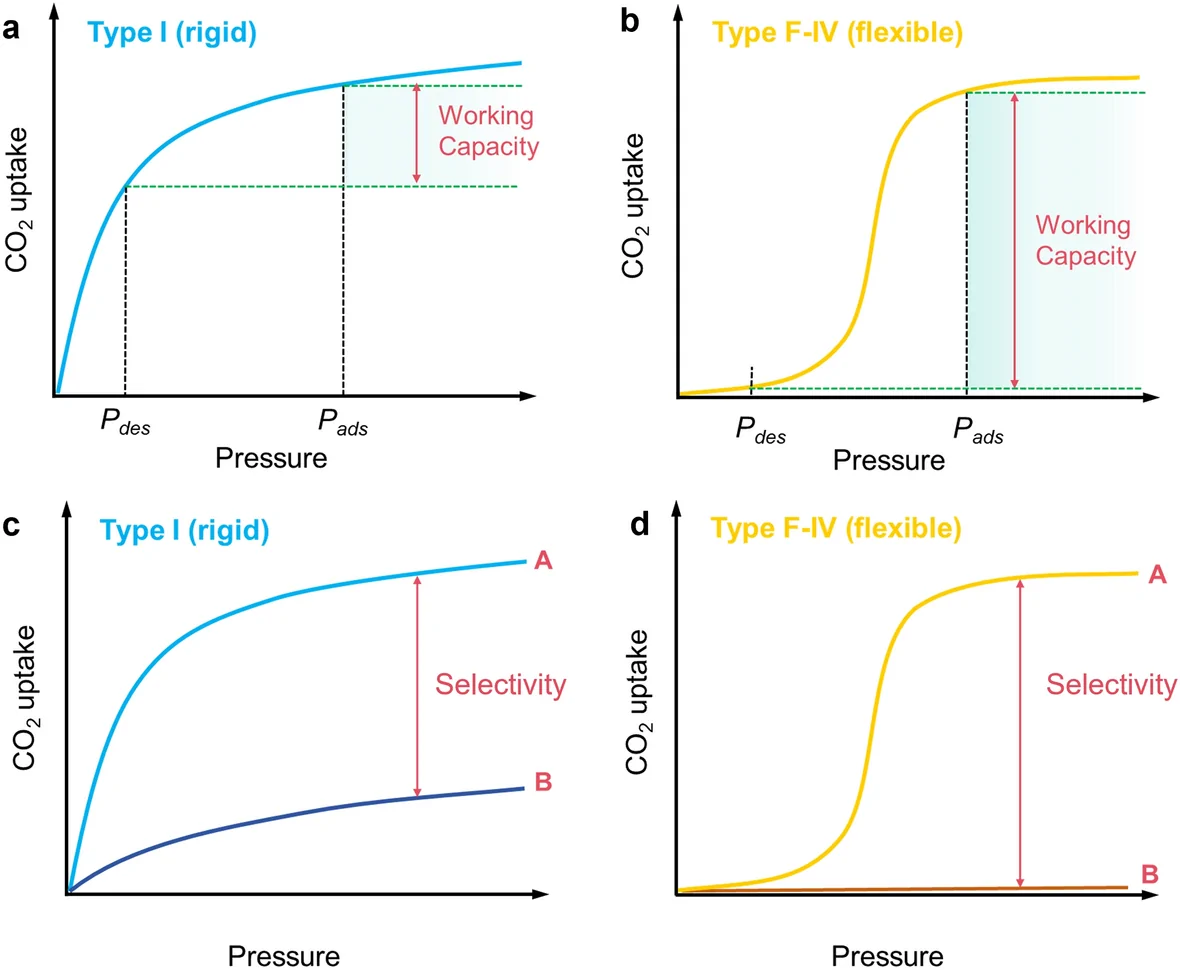
Gas adsorption isotherms for **a** and **c:** Type I (rigid); **b** and **d:** Type F-IV (flexible). **a** and** b** are single components; **c** and** d** are two components

When evaluating all CO_2_ adsorbents, adsorption capacity and selectivity are the primary indicators. However, considering practical applications, especially for MOF adsorbents, high working capacity and ideal adsorption selectivity are not only benchmarks for material performance but also key factors determining whether the material can transition from laboratory research to industrial application. It should be noted that equilibrium adsorption capacity and working capacity are central to gas adsorption. The former depends on the adsorbent’s pore structure, surface chemical properties, the nature of the adsorbate, and the target temperature and pressure. The working capacity refers to the disparity between the amount of gas adsorbed under target adsorption pressure and the quantity of gas remaining adsorbed at the lowest desorption pressure acceptable for system operation. Rigid microporous adsorbents typically exhibit characteristics of Langmuir-type (Type I) adsorption isotherms (Fig. 18a). Their limitation is that the adsorption or desorption process occurs outside the operating parameters, resulting in undesirable working capacity. Flexible adsorbents exhibit step-type or S-shaped adsorption isotherms at specific pressures or temperatures due to the breathing or gate-opening effect. This unique structural transformation behavior offers the potential to achieve high working capacities. As illustrated in Fig. 18, an ideal flexible MOF should maintain closed pores (or low adsorption capacity) under adsorption pressure (*P*_ads_) until the CO_2_ partial pressure reaches a critical point [61, 141, 142]. The pores open at this moment due to a structural change, which sharply increases the adsorption capacity. Hence, the difference in adsorption capacity between adsorption and desorption pressures increases. It also means that during desorption, a lower pressure is not required to release most of the CO_2_, which directly correlates to lower energy consumption. Besides, the selectivity of adsorbents for gases is mainly calculated through the Ideal Adsorption Solution Theory (IAST). For flexible MOFs, their selective mechanism goes beyond thermodynamic equilibrium, as rigid MOFs rely on, to include electrostatic forces and van der Waals force differences. Flexible MOFs can achieve excellent selectivity in molecular recognition. The core is that the structural transformation of flexible frameworks has a gating effect, and only when the physical and chemical properties (size, polarity, quadrupole moment, etc.) of the target molecules can provide sufficient energy drive (such as interaction with framework sites), can the opening of the structure be triggered.

However, how we can ensure that these flexible materials maintain high working capacity and selectivity during CO_2_ adsorption and separation remains an open question. Several key challenges must be addressed. First is the precise control of the gate-opening pressure (*P*_gate_) for flexible MOFs. As discussed before, high capacity is only demonstrated in practical applications when *P*_gate_ falls within the adsorption pressure range. Particularly in VPSA processes, the adsorbents capture target gas at a higher adsorption pressure (*P*_ads_) and regenerate at a lower desorption pressure (*P*_des_). For an ideal flexible MOF, its *P*_gate_ must be precisely controlled within the operating range of the adsorption tower, satisfying: *P*_des_ < *P*_gate_ < *P*_ads_. However, achieving this goal while ensuring stability during cycling remains a core challenge in current research. *P*_gate_ is influenced by multiple synergistic factors, including ligand flexibility, guest-framework interactions, and lattice energy. *P*_gate_ thermodynamically corresponds to the free energy change associated with structural phase transitions in the framework, exhibiting extreme sensitivity to the material’s microstructure. While effective, its regulation through metal selection, post-synthesis modification, or mixed ligand strategies involves complex processes and may induce unintended phase behaviors. Secondly, in order to use flexible MOFs for the separation of CO_2_ and mixed gases, the partial pressure of CO_2_ must exceed the gate pressure. Therefore, during the penetration process, a “slipping off” effect will occur, resulting in an inability to obtain high-purity product gas [73]. At present, multiple examples of this phenomenon have been reported, such as the breakthrough curves experiment on CH_4_/CO_2_ in MIL-53 [51] and the penetration experiments on CID-5/6 in CH_4_/C_2_H_6_ and CH_4_/CO_2_ [88]. In the VPSA section, we also discussed the slip phenomenon of ELM-11. Possible solutions include increasing the partial pressure of CO_2_ in the feed gas and mixing with rigid MOFs for sample loading.

### Potential Applications of Flexible MOFs for CO_2_ Capture in VPSA

**Fig. 19:**
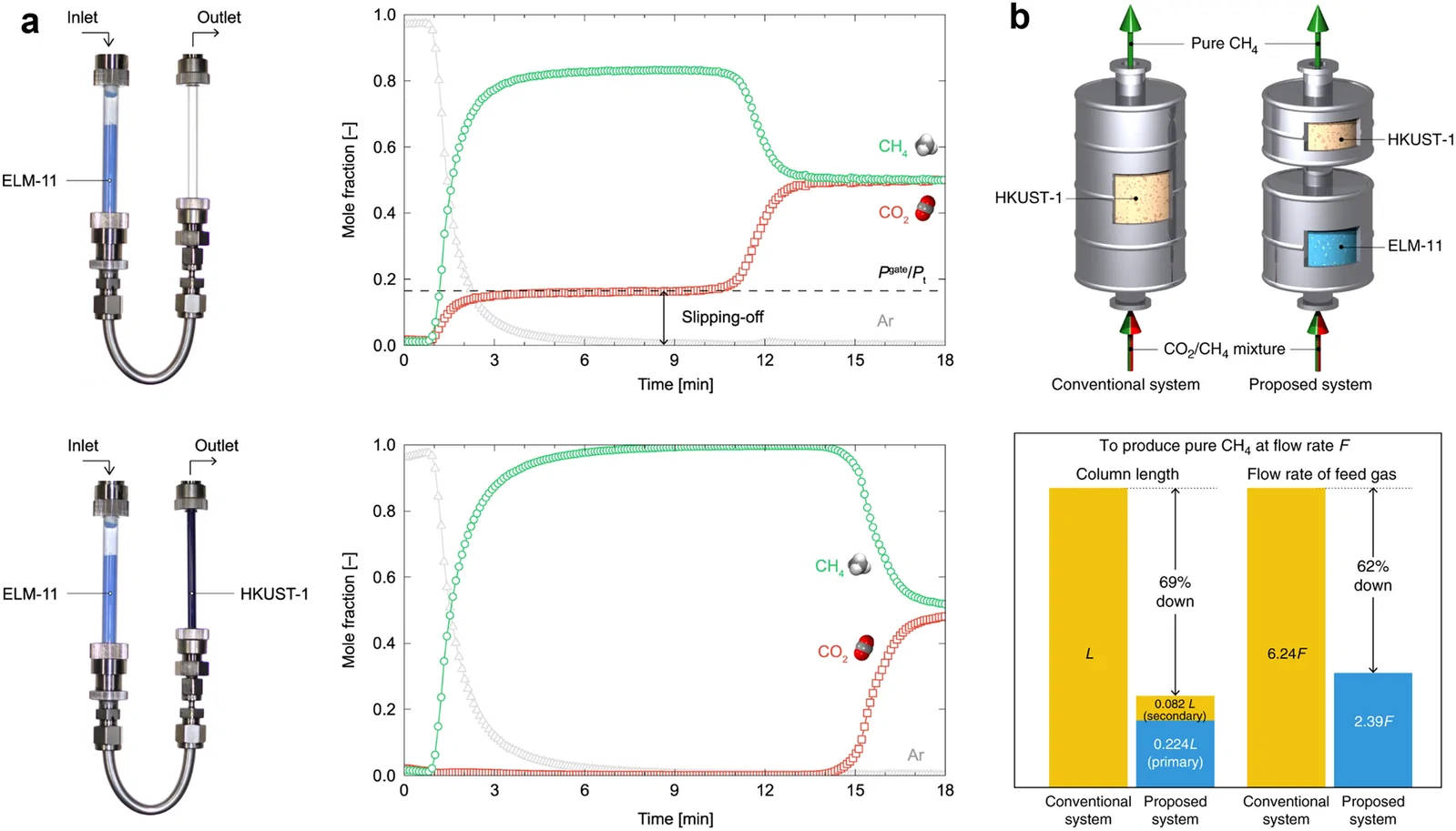
**a** Images of column systems and breakthrough curves for single-column (ELM-11) and sequential-column (ELM-11 + HKUST-1) systems. **b** Comparison between the two systems. Reproduced with permission from Ref. [73]. Copyright 2020, The Authors, under CC BY 4.0

VPSA is a physical separation technology based on differences in the adsorption capacity of gas molecules on adsorbent surfaces. Under periodic pressure changes, when mixed gas containing CO_2_ flows through the adsorbents, they achieve gas adsorption and separation due to differences in adsorption affinity, adsorption kinetics, and adsorption capacity for different gases [143–146]. Moreover, since the adsorption capacity of adsorbents varies with pressure, under high-pressure conditions, the adsorbent exhibits a higher adsorption capacity for certain gases (such as CO_2_). Conversely, under low-pressure or vacuum conditions, these gas molecules desorb from the adsorbent [6, 147, 148]. By periodically applying pressure swing, the adsorption and desorption cycles of the adsorbents can be achieved. The adsorbents used in VPSA primarily rely on van der Waals forces as the primary adsorption mechanism for capturing and separating CO_2_, such as carbon materials and zeolites. They typically show Langmuir-type adsorption isotherms [149, 150]. However, for actual VPSA systems, the high equilibrium adsorption capacities of these adsorbents do not necessarily result in high working capacities. Furthermore, desorption requires lower pressures for release, leading to higher energy consumption. In contrast, due to their unique gate-opening and breathing characteristics, flexible MOFs exhibit distinctive pressure-dependent adsorption behavior during CO_2_ pressure swing adsorption, resulting in step-like adsorption isotherms. Consequently, they demonstrate higher effective working capacity and selectivity compared to conventional adsorbents (Table 2). ELM-11, as a typical flexible MOF, exhibits gate-opening characteristics for CO_2_ adsorption that are highly compatible with the core requirements of VPSA systems, enabling efficient separation of mixed gases. In recent years, researchers have confirmed the applicability of ELM-11 in VPSA and its performance advantages through experimental verification and engineering design [73]. For the separation of landfill gas containing a 50:50 volume ratio mixture of CO_2_ and CH_4_, the objective is to achieve CH_4_ purification (purity ≥ 99.9%) and CO_2_ capture. Firstly, ELM-11 has the advantage of pressure-aided fast gating, which can adapt to the short-cycle operation of VPSA. At 273 K and 40.8 kPa CO_2_ pressure, the structural change of ELM-11 from cp to op can be completed within 10 s. When the pressure is increased to 250 kPa (298 K), 95% of the structural change requires only 1.5 s, significantly faster than the single-step operation time of fast VPSA. When CO_2_ pressure decreases at 2.4 kPa s^−1^, the phase transition from open to closed can be completed within 5 s, demonstrating significantly superior desorption kinetics compared to those of conventional rigid adsorbents. Secondly, the inherent thermal management capability of ELM-11 can alleviate the adiabatic temperature fluctuations of rapid VPSA. Research has shown that although HKUST-1 has a higher CO_2_ adsorption capacity under isothermal conditions, ELM-11 has superior CO_2_ adsorption capacity, selectivity, working capacity, and regeneration performance under adiabatic operating conditions due to its inherent thermal management ability. Therefore, evaluating the separation performance of flexible MOFs under adiabatic conditions requires consideration of not only adsorption isotherms, but also the net adsorption heat and the material’s intrinsic thermal management capability. Thirdly, the gating behavior of ELM-11 has CO_2_-specific recognition ability and high CO_2_ selectivity, and the “switching characteristics” of gate opening and closing make it perfectly regenerative, significantly improving the separation efficiency of VPSA. The open phase of ELM-11 can stably accommodate only CO_2_ (dipole interaction energy between CO_2_ and the framework is 25–26 kJ mol^−1^), while CH_4_ shows negligible adsorption due to weak interactions (< 20 kJ mol^−1^). At 298 K, ELM-11 achieved a CO_2_/CH_4_ selectivity of 82.3 for a 50:50 mixture, which is 9.5 times higher than HKUST-1 (8.67), and can reduce VPSA energy consumption for rinsing and purging. Lastly, the series system proposed by Shotaro Hiraide et al., featuring an “ELM-11 primary column + HKUST-1 secondary column,” resolves the slipping-off phenomenon commonly observed in flexible MOFs (Fig. 19a, b). This phenomenon occurs when the partial pressure of CO_2_ in the mixed gas falls below the threshold pressure, preventing the adsorbent from capturing CO_2_, resulting in CO_2_ loss alongside CH_4_. This provides a viable solution to eliminate the slipping-off effect in VPSA and serves as a reference for the application of flexible MOFs in practical separation engineering.

**Table 2:** Comparison of CO_2_ adsorption performances of different absorbents in (V)PSA process

Adsorbents	CO_2_ concentration (%)	T (K)	Adsorption capacity (mmol g^−1^)	Working capacity (mmol g^−1^) (0.2—1 bar)	Mixture Gas(v/v)	Process	Recovery (%)	Purity (%)	References
ZIF-7	15	298	2	1.4	CO_2_/CH_4_	PSA	72	99.9	[134]
ELM-11	15	298	3.1	3.1	CO_2_/N_2_	Four-step VPSA	67.8	99.7	[151]
ZnDatzBdc	20	298	2.2	2.1	CO_2_/N_2_	–	–	–	[65]
ELM-11	20	298	3.5	3.5	CO_2_/CH_4_	rapid VPSA	–	–	[73]
MIL-53(Al)	16	298	2.7	0.7	CO_2_/N_2_	two-step VPSA	54	39	[152]
F4_MIL-140A(Ce)	25.5	298	2.5	0.5	CO_2_/N_2_	Four-step VPSA	70	90	[147]
MIL-160	15	293	3.7	1.9	CO_2_/N_2_	Three-bed six-step VPSA	75.3 ± 2.0	95.3 ± 3.4	[153]
Mg-MOF-74	15	303	8.1	2	CO_2_/CO	Five-step VPSA	94.8	95.3	[154]
HKUST-1	20	298	9.1	8.1	CO_2_/CH_4_	rapid VPSA	–	–	[73]
Zeolite 13X	15	298	5.2	1.4	CO_2_/N_2_	Four-step VSA	86.4 ± 5.6	95.9 ± 1.1	[155]
BFS-13X	20	298	5.4	1	CO_2_/N_2_	two-step VPSA	58.7	62.2	[156]
Zeolite 13X	15	298	4.2	1.3	CO_2_/N_2_	Four-step VPSA	55.9	99.3	[151]
Zeolite NaX	15	298	4.2	1	CO_2_/N_2_	two-bed six-step VPSA	96.6	88.9	[157]
Activated Carbon	15	303	1.9	1.5	CO_2_/N_2_	two-step VPSA	74.7	95.3	[158]
PMC 1	50	298	3.1	0.8	CO_2_/CO	Two-step PSA	–	99.9(CO)	[159, 160]

**Fig. 20:**
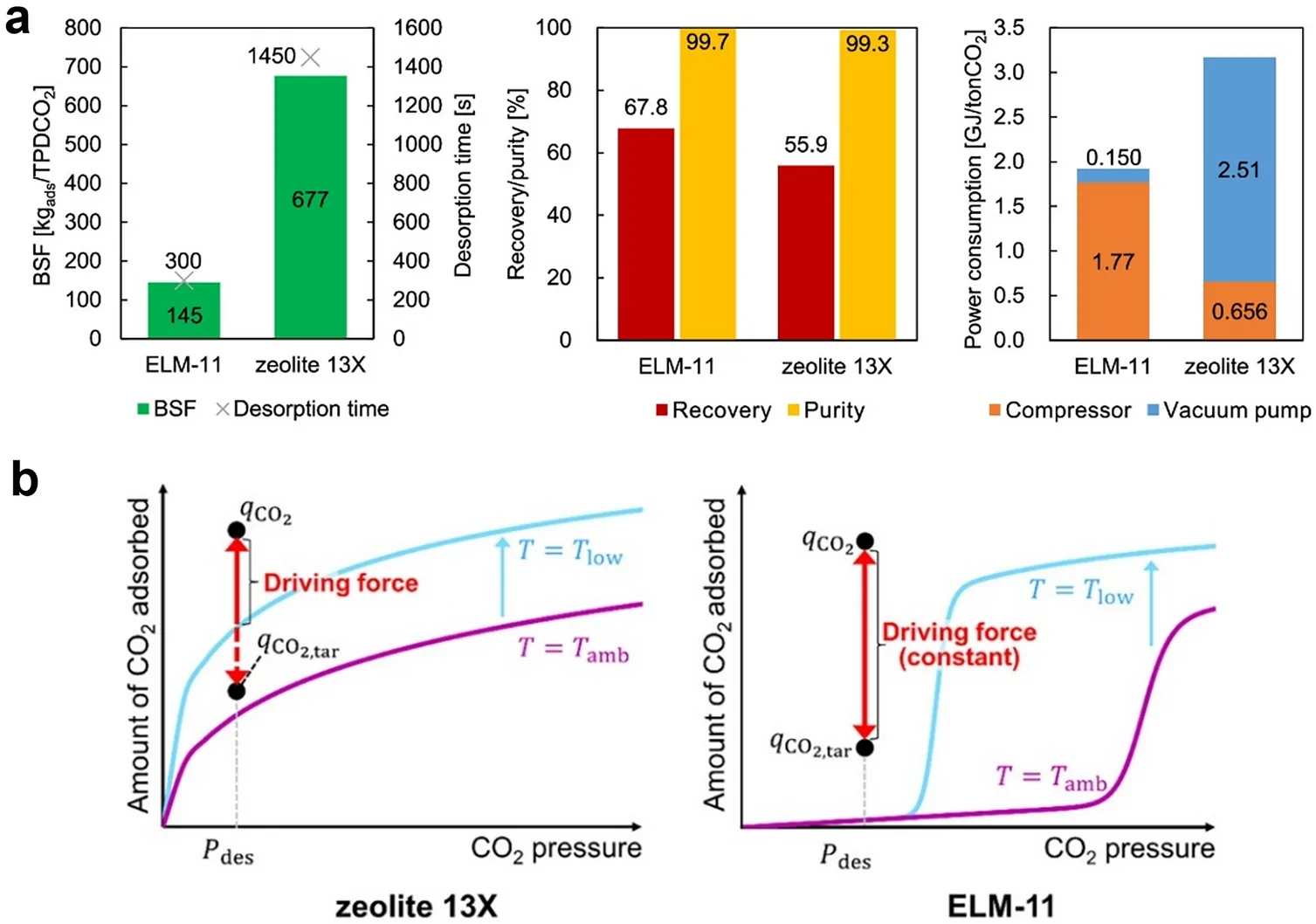
**a** Comparison between the adsorbents of ELM-11 and zeolite 13X. **b** The effects of temperature change on desorption time. Reproduced with permission from Ref. [151]. Copyright 2022, The Authors, Published by American Chemical Society

The dynamic simulation of adsorption processes, including full cycles of adsorption and desorption, represents another critical challenge for flexible MOFs. The detailed evaluation of dynamic simulation is crucial for estimating CO_2_ separation capacity and costs in the VPSA process, but the complicated dynamic behavior of flexible MOFs limits further analysis. Previously, researchers such as Remy et al. [161] and Hefti et al. [162] have simulated S-shaped adsorption isotherms. However, few studies have investigated adsorption processes that account for hysteresis effects. Yuya Takakura et al. [151] proposed a novel numerical method to address the S-type isotherm hysteresis challenge in flexible MOFs during simulated VPSA processes. By designing a single-bed four-step VPSA cycle, they simulated the adsorption process of ELM-11. Furthermore, sensitivity analysis of feed pressure and temperature determined the optimal operating strategy. Additionally, comparisons were made with the common carbon dioxide adsorbent 13X to confirm the advantages of ELM-11(Fig. 20). This study pioneered the research paradigm of “flexible MOF isotherm modeling-dynamic process simulation-performance optimization,” providing critical methodological support for the subsequent transformation of flexible MOFs from material preparation to engineering applications. The most apparent feature of flexible MOFs is that, when the framework responds to external stimuli, the adsorption capacity for gases can increase significantly. The excellent selectivity of such materials makes them highly promising for gas separation [163]. Nevertheless, the application research of MOFs has been limited by the absence of an appropriate isothermal model to precisely characterize the sudden shift in adsorption capacity. Therefore, for the simulation of flexible MOFs in the VPSA process, Eric F. May and his team [76, 164] focused on three types of flexible MOFs: Fe(bdp), Co(bdp), and ZIF-7, and explored the temperature dependence of their structural transformation, proposing a new LJMY-Langmuir isotherm model. It can precisely analyze the critical transition pressure (*p*_tr_), finite transition width (*σ*), and degree of hysteresis (*δp*_tr_) during adsorption–desorption processes. And it solves the problem that traditional models cannot accurately describe sudden changes in adsorption capacity.

**Fig. 21:**
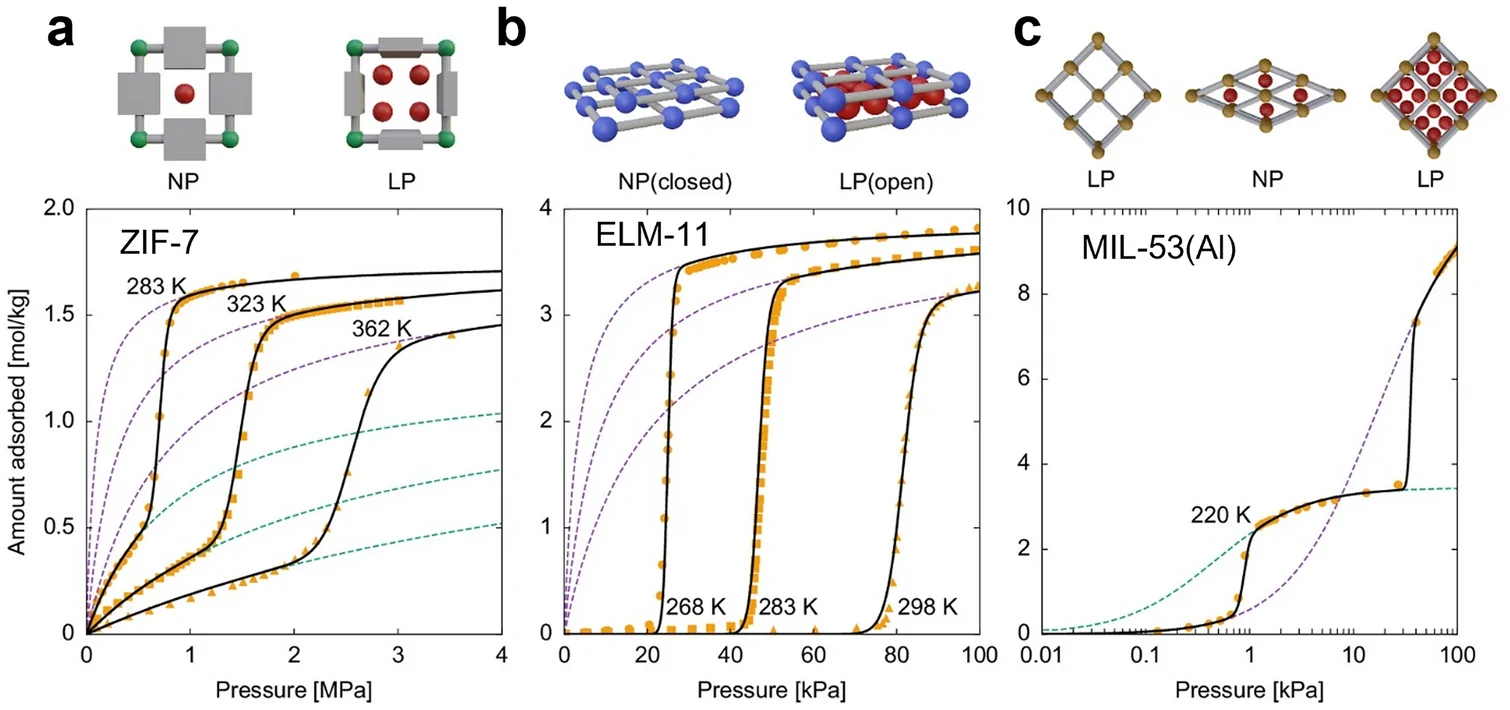
Function fitting results of **a** ZIF-7 for CH_4_, **b** ELM-11 for CO_2_, and **c** MIL-53(Al) for Xe. Orange, green, purple, and black are experimental data, *L*_*NP*_*, L*_*LP*_, and *N*, respectively. Reproduced with permission from Ref. [165]. Copyright 2023, The Author(s). (Color figure online)

Traditional simulation methods rely more on empirical equations and struggle to establish the internal connection between adsorption behavior and structural transformations. Hiraide et al. proposed the structural transformation adsorption isotherm equation (STA equation) based on statistical mechanics derivation, providing the first theoretically grounded model with clear physical significance for simulating CO_2_ adsorption isotherms in flexible MOFs [111]. This equation serves as the standard form for structural transition adsorption isotherms, much like the Langmuir equation represents Type I isotherms. By bridging the Langmuir isotherms of the np phase and the lp phase through the S-type function *σ(P)*, it can quantitatively describe CO_2_-induced structural transition processes. For example, when simulating the CO_2_ adsorption isotherms of ELM-11 (gate-opening) and MIL-53(Al) (breathing) CO_2_ adsorption isotherms (Fig. 21), it not only accurately reproduces the step positions and steepness of S-shaped isotherms at different temperatures but also correlates adsorption heat with the material’s inherent thermal management capability by introducing the host internal energy dissipation term Δ*U*_host_ and entropy change term Δ*S*_host_. Furthermore, the STA equation can be extended to integrate other isothermal models, such as Sips and BET, enhancing the fitting accuracy for complex pore-structured MOFs. This further advances the development direction of flexible MOFs in industrial adsorption and separation applications.

## Challenges and Prospects

### Adsorption Performance and Stability

The development of MOFs for CO_2_ capture with high capacity and good stability under practical conditions remains challenging. Specifically, when applied to realistic scenarios, most of them still suffer from low capacity at low CO_2_ concentrations (e.g., in flue gas or direct air capture), slow kinetics, and potential structural collapse [166]. One of the most critical challenges is the presence of H_2_O in practical gas streams. They often severely compete with CO_2_ for open metal sites or functional groups, leading to a drastic decrease in CO_2_ uptake. Moreover, the attack of water molecules can cause the hydrolysis of coordination bonds, resulting in the structural collapse of MOFs. Meanwhile, the weak host–guest interaction under ultra-low CO_2_ concentration further leads to poor selectivity over non-target components such as N_2_. In some specific industrial carbon capture contexts, such as the processing of blast furnace gas in the steel industry, the co-existence of significant CO concentrations with CO_2_ demands efficient CO and CO_2_ separation to facilitate CO reclamation and enhance its economic reutilization value. This results in a high purity of the adsorbed target gas. It also arises from the mixed adsorption of CO_2_ molecules with very similar sizes, which can easily lead to co-adsorption [167–169]. In addition, some sulfur-containing gas components will also compete with CO_2_ for adsorption or destroy the active sites on the surface of the adsorbent. Therefore, considering the thermodynamic stability of the framework is a key part in achieving ideal performance [170]. In future research, potential solutions include introducing hydrophobic microenvironments (e.g., fluorinated or alkyl functional groups) into the pores to shield the CO_2_-binding sites from water interference, or constructing multi-variate MOFs to balance hydrophobicity and CO_2_ affinity [171]. Therefore, the design of a robust system with both a high CO_2_ capacity at low concentrations, excellent stability, and regulated porosity is a worthwhile direction to explore for practical applications.

### Scalable Preparation and Shaping

In laboratory studies, the performance of flexible MOFs is typically evaluated using micron-sized crystalline powders synthesized via mild, refined solvothermal methods. However, industrial adsorption separation processes require adsorbents to possess specific macroscopic morphologies (e.g., spherical particles, extruded rods, or honeycomb monoliths) to achieve low pressure drop, high mechanical strength, and good mass transfer performance within adsorption columns. The process of converting laboratory microcrystalline powders into industrially viable shaped samples presents challenges for flexible MOFs, including reduced adsorption performance and compromised flexibility [172, 173].

Currently, companies in multiple countries, including the USA, Canada, the UK, Germany, and China, are advancing MOFs from the laboratory to industrial applications. They are testing adsorbents in flue gases from coal-fired power plants, waste incineration facilities, and other industrial sources, targeting lower energy consumption and higher moisture stability for large-scale CO_2_ capture. CALF-20 is an adsorbent that combines high capacity, high selectivity, high stability, and scalability, offering a low-energy, durable, and industrially viable solution for carbon dioxide capture technology. The study also demonstrates that MOF materials have progressed from laboratory research to industrial-scale testing [174]. Since January 2021, CALF-20 has been undergoing industrial trials at the Lafarge-Holcim cement plant in Canada, capturing 1 ton of carbon dioxide per day. It is also the world’s first industrially demonstrated MOF material. Approximately 38% of CALF-20’s structure consists of pore channels, with each gram possessing a specific surface area of 528 m^2^ of pore volume. It exhibits a high CO_2_ adsorption amount above room temperature. CALF-20’s adsorption capability and selectivity far surpass traditional materials, functioning like a “molecular cage” tailored for CO_2_. Additionally, commercial entities, including BASF, Nuada, and Framergy, have successfully synthesized various solid MOF materials, including ZIF-8, MIL-53(Al), MOF-74, HKUST-1, and MIL-101(Fe) [173, 175]. Despite these achievements, only a handful of flexible MOF products have been scaled up for industrial applications. Increasing collaborations are emerging between fundamental science and technology and the commercial sector.

Over the past decade, researchers have devoted considerable effort to developing novel shaping methods of MOFs to promote their practical applications [176]. The in situ crystallization molding method is a one-step molding method in which the substrate material is immersed in a reaction solution containing metal and ligand, and the MOF crystal is formed on the substrate. This method is often used to prepare membrane materials [177]. Mechanical shaping involves adding powdered material to a mold and applying mechanical pressure to compress it into bars or blocks. This method is simple to operate and offers good material stability [178]. Hot pressing is also a convenient, efficient, and environmentally friendly method that has been successfully applied to the synthesis and shaping of various MOFs and carbon-organic frameworks (COFs) [179, 180]. Furthermore, the binder-mediated shaping method can prepare MOF products in desired shapes. By selecting suitable binders, it can also modify the physical properties and hydrophobicity of MOFs while enhancing their stability [181, 182]. These shaping methods have extensively promoted the transition of MOF adsorbents from laboratory research to industrial applications.

**Fig. 22:**
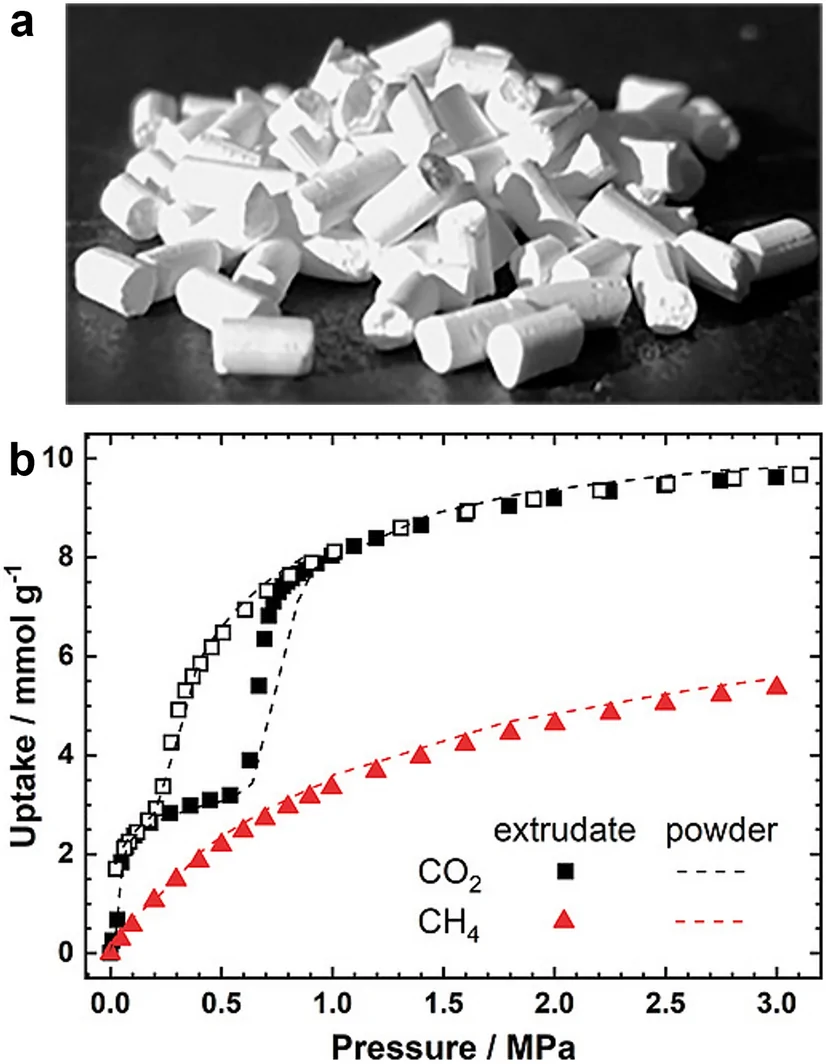
**a** Image of as-prepared MIL-53(Al) extrudates. **b** CO_2_ and CH_4_ sorption isotherms of MIL-53 extrudates and MIL-53 powder. Reproduced with permission from Ref. [173]. Copyright 2019, The Authors, Published by Wiley–VCH

Two aspects should be considered in molding a flexible adsorbent: the dynamic structure must not be damaged under external mechanical stress, and the molding process and binder can improve stability while maintaining adsorption performance. Some particles failed to withstand the mechanical stress induced by phase transitions during cyclic adsorption–desorption, fracturing upon exposure to high-pressure gas during adsorption. Therefore, the exploration of molding processes (sol–gel method, plasma-assisted technology, deep eutectic solvents, etc.) and the selection of binders (such as polyvinyl alcohol (PVA), polyvinylidene fluoride (PVDF), methyl cellulose, etc.) are very important for improving the stability of the flexible adsorbent. MIL-53, a typical example, in which researchers reported a simplified extrusion molding method using methylcellulose (MC) as a binder, successfully yielded flexible MOFs such as MIL-53 and MIL-53-NH_2_ [173]. The material exhibits enhanced macroscopic mechanical stability while fully retaining its framework flexibility. Particles containing 5% binder demonstrate optimal mechanical stability, reducing pore volume loss more effectively than conventional PVA. In situ XRD confirms that under specific humidity conditions (20%–45% relative humidity (RH)), MIL-53 extrudates maintain breathability during CO_2_ adsorption, with phase transition kinetics similar to those of the powder. Furthermore, high-pressure adsorption experiments demonstrated that the extrudates’ adsorption capabilities for CO_2_ and CH_4_ were similar to those of the powder samples. (Fig. 22) All molded samples exhibited stable binder behavior without decomposition at regeneration temperatures ranging from 100 to 200 °C. However, another study found that high pressure and temperature affect the flexibility of MIL-53(Al). These conditions influence the interaction between CO_2_ and the aluminum and hydroxyl groups within the framework, thereby increasing adsorption capacity through chemisorption [183]. Kundu et al. investigated the effect of introducing hydroxyl groups into the ligands of MIL-53(Al). The molded samples exhibited minor changes in adsorption isotherms but fractured due to the inability to withstand the adsorption force [48].

**Fig. 23:**
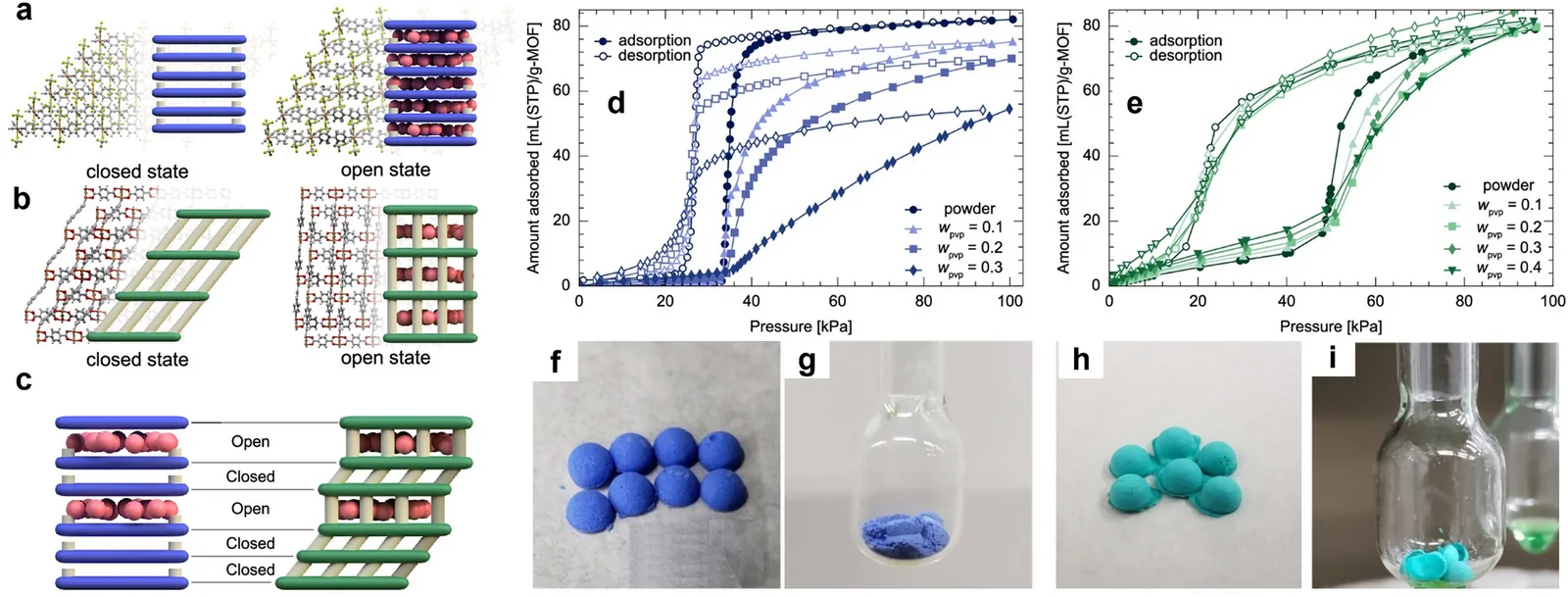
**a** Crystal structures of ELM-11. **b** Atomistic structures of JG-MOF. **c** Schematic diagrams of particle layer-by-layer structural transformation. CO_2_ adsorption isotherms: **d** on molded ELM-11 at 273 K and **e** on molded JG-MOF at 263 K. Images of the ELM-11 pellet and JG-MOF pellet: before (**f** and **h**) and after (**g** and **i**) CO_2_ adsorption. Reproduced with permission from Ref. [188]. Copyright 2023, American Chemical Society

Previous studies have shown that external forces applied by the binder during shaping can disrupt the gated adsorption properties of flexible MOFs, leading to a flattening of the adsorption step (i.e., the “slacking phenomenon”) [184–186]. However, the mechanism and regulation methods for this phenomenon remain unclear. Hiraide’s team investigated the slacking phenomenon in the gate adsorption behavior of the flexible MOF ELM-11 under external force [187]. The gated adsorption behavior of ELM-11 particles prepared with different concentrations of PVP binder was investigated. Combining GCMC simulations and free energy analysis, two core factors of the relaxation mechanism were revealed: asynchronous interlayer deformation in ELM-11 particles and nonlinear external force potential energy. Notably, the free energy analysis also found that external forces broaden the hysteresis loop in gated adsorption. Ultimately, a “core–shell-like” shaped method was proposed to optimize the shaping strategy for flexible MOFs. In subsequent studies, they comprehensively validated and extended the gate adsorption slacking mechanism from both theoretical and experimental perspectives [188]. Moreover, the correlation between volumetric expansion rate and relaxation degree was clarified. Free energy analysis revealed that for ELM-11 with a high expansion rate (30%), the powder step was distinct, and significant slacking occurred after forming. Furthermore, stronger external forces (e.g., higher binder content) led to more pronounced slacking (Fig. 23). In contrast, for JG-MOF with a low expansion rate (10%), the adsorption isotherms between powder and formed particles showed minimal differences, maintaining structural stability even after multiple adsorption cycles. Therefore, exploring forming methods that reduce the impact of external forces on flexible MOF particles is a crucial part of advancing sustainable MOF-based material technologies.

### Functional Integration

MOFs have emerged as one of the most competitive core materials in CCUS technology. Flexible MOFs with single adsorption functions are increasingly unable to meet the demands for efficient carbon processing under varying conditions. Functional integration or composite materials offer new pathways for breakthroughs in CO_2_ adsorption and separation technology. Current research on flexible MOFs primarily focuses on optimizing single-adsorption performance. Looking ahead, precise structural design offers a promising route toward multifunctional integration — coupling adsorption with catalysis, separation-storage, or real-time detection. Numerous studies have successfully demonstrated the integration of CO_2_ capture and catalysis within MOF frameworks [189, 190]. Hence, developing integrated flexible MOFs to achieve self-adapting CO_2_ capture and conversion is a promising direction for future research [191, 192]. When dealing with CO_2_ emissions from various industrial sectors, a single adsorbent struggles to meet the diverse separation requirements. For instance, nuclear power plant operations generate ^13^C and ^14^C, which exist in the exhaust gases as ^14^CO_2_ and ^14^CO [11, 193, 194]. MOF materials can effectively adsorb and separate these gases, facilitating CO_2_ capture and simultaneous purification of pollutants. Functionally integrated MOFs can achieve precise matching of extreme separation requirements through the synergistic integration of multiple functional modules. The atomically regulated pore structure realizes the quantum sieving effect for isotope molecules with extremely small size differences. Furthermore, the structural flexibility of the material can couple with the radiation field, enabling in situ regulation and adaptive control of adsorption behavior. Traditional rigid porous materials mainly rely on the quantum sieving effect to achieve CO_2_ isotope separation, but their fixed pore structure is difficult to precisely match the tiny kinetic diameter difference between CO_2_ isotopologues, resulting in a bottleneck in both separation selectivity and working capacity. The core advantage of flexible MOFs in CO_2_ isotope separation lies in their dynamically tunable gate-opening behavior, which can achieve precise differentiation of CO_2_ isotopologues based on extremely small differences in adsorption thermodynamics and kinetics. For example, Gu et al. reported two flip-flop dynamic crystals (FDCs) for H_2_O and its isotopes (H_2_O/HDO/D_2_O) separation [195], and laid a solid research foundation for the development of this field. Nevertheless, there are still non-negligible challenges for the practical application of functionally integrated MOFs in nuclear industry isotope separation. First, the structural durability and adsorption performance retention of MOFs under high-dose irradiation. In addition, the anti-interference ability of functional integrated MOFs to coexisting impurities needs to be improved. This MOF design, which integrates functions such as selective CO_2_ separation and in situ conversion, will contribute to the development of next-generation carbon capture materials. Exploiting dynamic structural responses for molecular-level recognition and multifunctional integration holds great promise for addressing industrial separation challenges within CCUS, including flue gas capture and direct air capture (DAC).

### Composite Adsorbents

The development of MOF-based composite adsorbents represents a pivotal strategy for optimizing CO_2_ adsorption and separation performance, with diverse composite systems including MOFs/MOFs, MOFs/carbon materials, and MOFs/zeolites showing great promise due to their synergistic effects [196–199]. Integrating MOFs of complementary properties, such as flexibility and CO_2_ selectivity paired with thermal stability and mechanical robustness, enables the construction of composite frameworks with synergistic adsorption behavior. For example, integrating a flexible MOF with a rigid MOF can address the poor stability of single flexible MOFs under harsh conditions while retaining their high capacity and selectivity for CO_2_ adsorption. This also works to resolve the slipping-off problem in VPSA [73]. Additionally, by combining rigid MOFs with macroporous/mesoporous structures and microporous flexible MOFs, a hierarchical pore system can be constructed. This structure not only accelerates the diffusion kinetics of CO_2_ molecules but also enhances CO_2_ affinity at low pressures through the pre-adsorption effect of the rigid layer. Meanwhile, the internal flexible layer provides substantial storage capacity via structural phase transitions at high pressure, resulting in outstanding adsorption performance across a wide pressure range. The carbon-based component enhances the mechanical strength, thermal stability, and mass transfer efficiency of the hybrid adsorbent through interconnected pore channels. These structural features are critical for sustaining rapid CO_2_ adsorption–desorption cycles in industrial applications. Furthermore, the conductive nature of carbon materials enables the development of electro-responsive MOF/carbon composites, allowing for electrochemically modulated CO_2_ adsorption and desorption—an approach that can significantly reduce regeneration energy consumption compared to traditional thermal or pressure swing processes [200, 201]. MOFs/zeolites composites, as a classic composite system, integrates the uniform pore structure, excellent thermal stability, and strong water vapor resistance of zeolites with the high specific surface area, structural flexibility, and tunable CO_2_ sites of MOFs. Interface engineering strategies (e.g., in situ growth, mechanical mixing, layer-by-layer assembly) can optimize interfacial compatibility between MOFs and zeolites, enabling synergistic regulation of pore size and active sites [202–204]. Collectively, the rational design and fabrication of MOF-based composite adsorbents hold the key to overcoming the performance limitations of single-component adsorbents. Future research should focus on clarifying the interfacial interaction mechanisms within these hybrid systems, developing scalable and low-cost synthesis methods, and optimizing the synergy between components. Collectively, these efforts will drive the practical deployment of MOF-based composite adsorbents toward real-world CO_2_ capture and separation, ultimately advancing efficient and cost-effective CCUS technologies.

### Multi-module In-Situ Characterization

The intelligent adsorption behavior of flexible MOFs stems from their dynamic structural responses to external stimuli. However, conventional characterization methods predominantly focus on static structures before and after adsorption. Observing the critical phase transition processes during adsorption undoubtedly presents a new challenge for characterization techniques. The sustained and steady development of flexible MOFs depends heavily on the creation and application of sophisticated characterization methods, which are capable of capturing the real-time dynamic behavior of frameworks across multiple temporal and spatial scales under operational conditions. These characterization methods are not only essential for verifying observed phenomena but also for quantitatively understanding their dynamics and thermodynamics, thereby enabling precise modulation of material functionality.

**Fig. 24:**
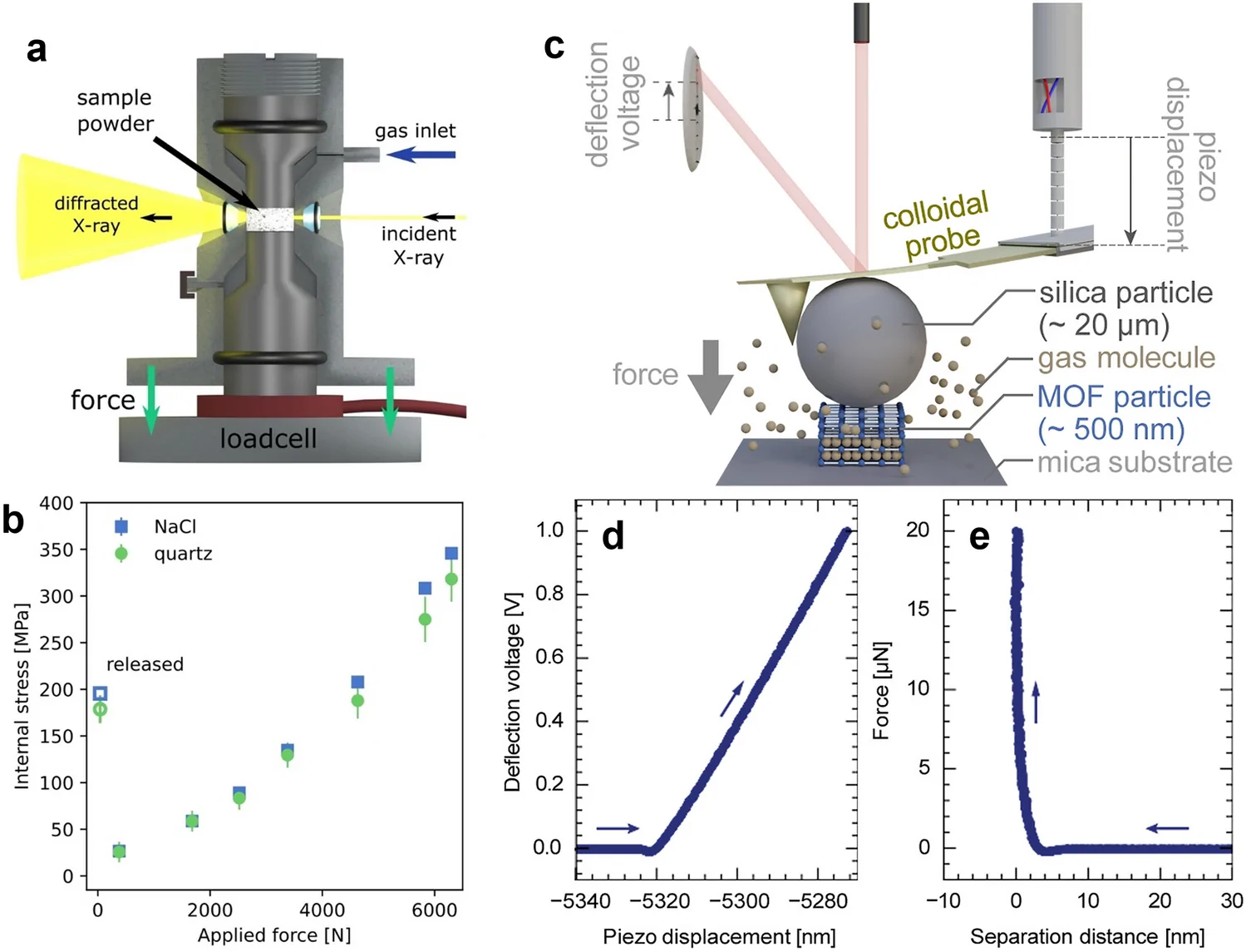
**a** Schematic diagram of the CSPC cell design. **b** Evolution of internal cell pressure versus applied force. Reproduced with permission from Ref. [206]. Copyright 2022, Wiley–VCH. **c** Schematics of the AFM measurement principle. **d** Piezo expansion/contraction and changes in the laser detection point caused by probe bending (displacement vs deflection voltage profile as raw data). **e** A separation distance vs force profile. **d** and **e** represent the results of pressing a mica substrate under an EtOH/N_2_ atmosphere. Reproduced with permission from Ref. [205]. Copyright 2025, American Chemical Society

Understanding the complete process of flexible MOFs, from local bond changes in unit cells to macroscopic crystal morphology alterations, requires a coordinated multiscale characterization approach. Firstly, dynamic structural analysis at the atomic level can be achieved through time-resolved synchrotron in situ XRD measurements, which reveal dynamic variations in the crystal structure during adsorption–desorption cycles [73]. Combining neutron powder diffraction with pair distribution function (PDF) analysis enables characterization of local structural changes in disordered or amorphous regions, identification of guest molecule adsorption sites (CO_2_ and H_2_O) within the framework, and elucidation of their roles in triggering phase transitions. Furthermore, phase transitions may not occur synchronously within a single crystal or between different crystals. In situ scanning electron microscopy and environmental atomic force microscopy(AFM) enable direct observation of crystal deformation, cracking, and other reactions during the adsorption of guest molecules (Fig. 24c-e). For instance, Watanabe and his team revealed the difference in flexible transitions between ELM-12 and DUT-8 (Ni) using atomic force microscopy combined with thermodynamic analysis [141]. This approach offered in-depth understanding into the transition mechanism and its effect on the integral adsorption isotherm, offering a novel veiwpoint on how the behavior of individual particles regulates the overall performance of the material [205]. In situ infrared or Raman spectroscopy can detect changes in molecular bond vibration frequencies during adsorption, thereby revealing the establishment and evolution of guest–host interactions. In situ solid-state NMR, particularly when combined with probes such as ^13^C and ^1^H, enables quantitative analysis of the occupancy distribution of adsorbed molecules across different sites, their dynamic behavior, and alterations in the local chemical environment of the framework. This provides molecular-level evidence for the energy barriers to phase transitions. Meanwhile, the structural evolution pathways and kinetic data obtained from in situ experiments provide a crucial foundation for molecular dynamics and Monte Carlo simulations [74, 187, 188]. Moreover, simulations can conversely reveal transient intermediate states and molecular-level mechanisms that are difficult to observe in experiments. Additionally, designing novel in-situ X-ray reaction cells is also an effective characterization method. Recently, Yot and his team designed a new pressure cell capable of a combined stress–pressure clamp (CSPC), successfully tracking the evolution of carbon dioxide breathing behavior in the MIL-53(Al) under mechanical compression [206] (Fig. 24a, b).

### Machine Learning-Assisted Design

**Fig. 25:**
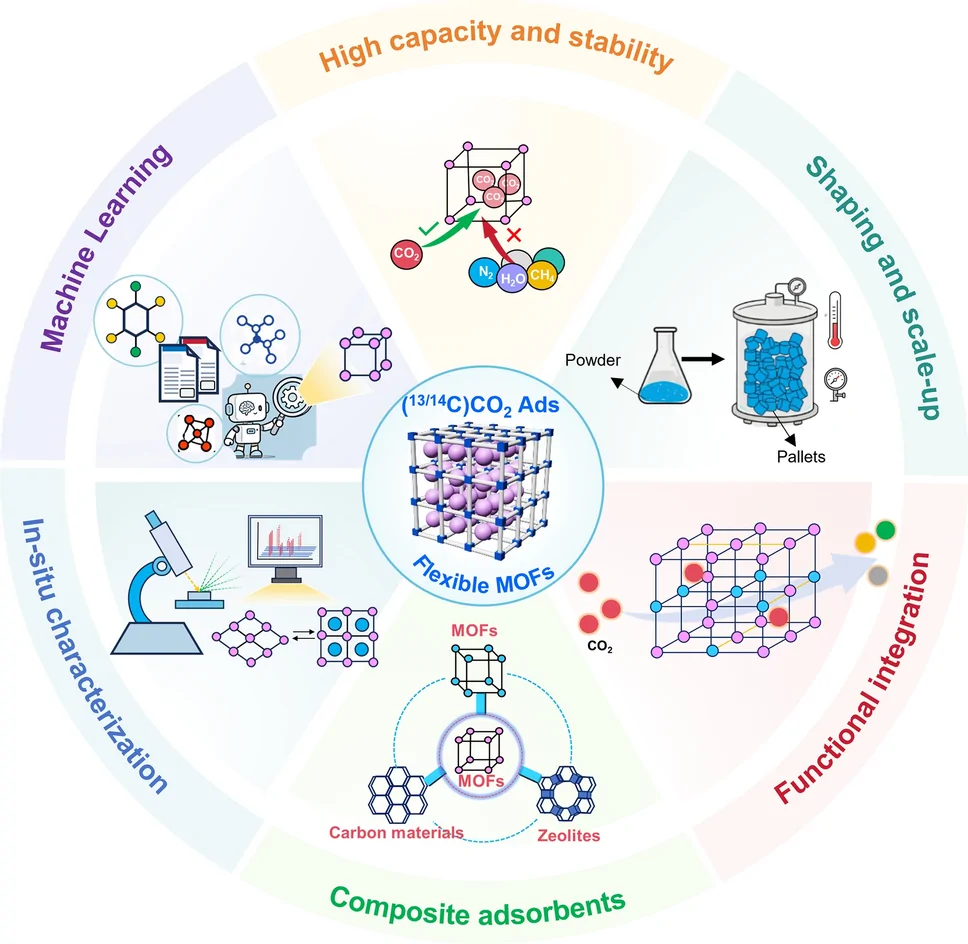
Challenges and perspectives of flexible MOFs for CO_2_ adsorption: high capacity and stability, shaping and scale-up, functional integration, composites adsorption, in-situ characterization, and machine learning-assisted design

In recent years, research on flexible MOFs in the field of adsorption has been steadily advancing. Systematic investigations of experimental methods can provide intuitive data for CO_2_ adsorption and separation. Meanwhile, flexible MOFs demonstrate irreplaceable advantages in various CO_2_ adsorption and separation scenarios due to their tunable and designable structures. However, traditional approaches show numerous uncontrollable factors from material design to achieving high-performance applications. The combination of machine learning (ML) and artificial intelligence (AI) with conventional methods has proven effective in predicting gas adsorption properties [207–210]. They have become powerful tools for effectively establishing multi-dimensional structure–property relationships, identifying key performance factors, and formulating design guidelines for target materials [211–214]. For example, Elkamel et al. used machine learning algorithm models to analyze CO_2_ adsorption data and parameters, evaluating the factors influencing adsorption capacity [215]. Li et al. explored the gas-separation performance of MOF-5 analogues using machine learning and verified the strategy experimentally [216]. Steckel et al. used machine learning to explore the influence of SIFSIX-3-Cu frame flexibility on CO_2_ adsorption [217]. Future work should establish a cross-scale multi-objective optimization framework, incorporating key CO_2_ adsorption parameters as optimization targets. Such models would enable rational prediction and control of core structural units, including ligand length and metal–ligand bonding strength. Specifically, by introducing process parameters (e.g., pressure swing adsorption conditions) as model inputs, reinforcement learning optimizes the alignment between material structures and operational parameters. For instance, it enables the targeted design of flexible MOFs featuring low gating pressure and high cycle stability. Consequently, by integrating multimodal simulation and experimental data with intelligent algorithms, this approach is expected to accelerate the industrial deployment of MOF adsorbents in carbon capture and provide critical material support for low-carbon targets (Fig. 25).
